# On Evaluating Black-Box Explainable AI Methods for Enhancing Anomaly Detection in Autonomous Driving Systems

**DOI:** 10.3390/s24113515

**Published:** 2024-05-29

**Authors:** Sazid Nazat, Osvaldo Arreche, Mustafa Abdallah

**Affiliations:** 1 Electrical and Computer Engineering Department, Purdue School of Engineering and Technology, Indiana University—Purdue University Indianapolis, Indianapolis, IN 46202, USA; snazat@iu.edu (S.N.); oarreche@iu.edu (O.A.); 2 Computer and Information Technology Department, Purdue School of Engineering and Technology, Indiana University—Purdue University Indianapolis, Indianapolis, IN 46202, USA

**Keywords:** anomaly detection, autonomous driving, explainable AI, Shapley additive explanations, LIME, feature extraction, VeReMi dataset

## Abstract

The recent advancements in autonomous driving come with the associated cybersecurity issue of compromising networks of autonomous vehicles (AVs), motivating the use of AI models for detecting anomalies on these networks. In this context, the usage of explainable AI (XAI) for explaining the behavior of these anomaly detection AI models is crucial. This work introduces a comprehensive framework to assess black-box XAI techniques for anomaly detection within AVs, facilitating the examination of both global and local XAI methods to elucidate the decisions made by XAI techniques that explain the behavior of AI models classifying anomalous AV behavior. By considering six evaluation metrics (descriptive accuracy, sparsity, stability, efficiency, robustness, and completeness), the framework evaluates two well-known black-box XAI techniques, SHAP and LIME, involving applying XAI techniques to identify primary features crucial for anomaly classification, followed by extensive experiments assessing SHAP and LIME across the six metrics using two prevalent autonomous driving datasets, VeReMi and Sensor. This study advances the deployment of black-box XAI methods for real-world anomaly detection in autonomous driving systems, contributing valuable insights into the strengths and limitations of current black-box XAI methods within this critical domain.

## 1. Introduction

In the realm of autonomous driving, autonomous vehicles (AVs) are equipped with sophisticated sensors, intelligent software, and advanced communication systems, allowing them to navigate and operate without human intervention [1]. The integration of various artificial intelligence (AI) models empower AVs to perceive their surroundings, identify objects, and make decisions autonomously. While AVs offer substantial advantages, a significant challenge arises from their susceptibility to cybersecurity threats, exposing them to various types of cyber-attacks [2]. Research in this domain has focused on addressing cybersecurity concerns, particularly in leveraging AI models to enhance the security of vehicular ad hoc networks (VANETs) [3]. Numerous studies have delved into securing VANETs by detecting and preventing different types of attacks or anomalies [4,5,6]. However, most of these works have used a few AI models to detect anomalous AVs [3,7,8] and focused more on the accuracy of anomaly detection models without further exploring the main factors that affect the decisions of AI models in detecting anomalies [9,10].

None of these studies have identified the key features crucial for effectively classifying anomalous AVs. Consequently, relying on such AI models, which play a pivotal role in making critical decisions for AVs, entails inherent risks. The challenge becomes particularly pronounced in the case of AI models employed in AVs, where understanding the rationale behind specific judgments made by these models is crucial. The intricacies of how an AI model functions and the factors it considers before arriving at an anomaly classification decision remain elusive [11]. Consequently, further exploration is needed to unravel how AI models detect anomalies in a VANET and the decision-making process that leads to classifying an AV as anomalous.

The field of explainable AI (XAI) holds the potential to address trust issues within the security of autonomous vehicle networks, such as VANETs [12]. XAI brings three key advantages when integrated into existing AI models for autonomous driving. Firstly, in cases where the AI model makes an incorrect anomaly prediction, XAI facilitates understanding the contributing factors, aiding in the prevention of similar issues in the future. Secondly, it fosters trust between human operators and the AI agent, particularly crucial in high-risk domains like AV security [13], by substantiating AI predictions with evidence. Thirdly, XAI contributes to enhancing the performance of anomaly prediction AI models by providing insights into why and how the models operate  [14,15]. This understanding enables AV manufacturers and scientists to pinpoint areas for fine-tuning and optimization, such as identifying the most crucial sensors on the AV. Thus, XAI serves to elucidate AI model decisions in a manner comprehensible to human operators, including safety operators.

Assessing the effectiveness of XAI methods for anomaly detection in autonomous vehicles is of paramount importance for various reasons. Firstly, given the notable cybersecurity threats and vulnerabilities associated with autonomous vehicles, including potential cyber-attacks that could manipulate navigation systems or sensors [16], a reliable XAI method is crucial. Such a method aids security experts in comprehending and validating decisions made by AI systems controlling autonomous vehicles [11]. By offering explanations for the classification of a vehicle’s behavior as anomalous, XAI empowers researchers to scrutinize the safety and security of autonomous systems. Additionally, establishing trust in these XAI methods for autonomous vehicles is imperative for human operators [17], considering the life-critical decisions made by these vehicles. Moreover, shedding light on potential shortcomings in existing XAI methods assists developers in identifying and addressing issues, such as understanding the factors that led to incorrect explanations by XAI methods, thereby pinpointing the causes of erroneous outcomes by XAI models [18]. Taken together, these crucial factors motivate the need of having a systematic framework for evaluating XAI methods for enhancing anomaly detection in autonomous driving.

The primary purpose of this paper is to explore such a gap in anomaly detection in autonomous driving. We propose a framework for evaluating black-box XAI methods (SHAP and LIME) that are used to interpret the decisions of black-box AI models, with the main focus on anomaly detection in autonomous vehicles. In our framework, we take into account different attack characteristics and various AI classification methods in order to distinguish between normal and anomalous AVs. We process the raw data for each AV and extract basic features that can be leveraged to detect anomalies. In addition, we make use of two popular black-box XAI methods, Shapley additive explanations (SHAP) [19], and local interpretable model-agnostic explanations (LIME) [20] to visualize the primary features that the AI models are employing in order to categorize different AVs. We then extract the main features that identify whether an AV is anomalous or not.

To thoroughly assess the utility of these two popular XAI techniques, we evaluate them based on six key performance metrics: descriptive accuracy, sparsity, stability, efficiency, robustness, and completeness. In particular, we build on the prior work [21] that describes six metrics to evaluate XAI methods. The evaluation metrics are briefly explained as follows:

**(1)**  **Descriptive accuracy:** This metric quantifies the alignment between feature importance assigned by the XAI technique and the true impacts of features on the AI model’s predictions. It is measured by systematically removing top features and assessing degradation in the predictive performance of the AI model.
**(2)**  **Sparsity:** This metric assesses whether the importance of explaining a model’s logic is spread out or concentrated among its features. If the explanations are sparse, it means only a few features play a crucial role in the model’s decision making. For instance, if 8 out of 10 features related to anomaly detection in an autonomous driving system are below a small threshold (close to zero), it implies that only 2 features hold a significant influence on the model’s decision making. Consequently, the sparsity is high. Utilizing an XAI method with high sparsity in autonomous driving monitoring can assist analysts in monitoring AV networks by focusing on a smaller set of critical features.
**(3)**  **Stability:** The stability metric measures how consistent the XAI method is in generating its explanations. Higher stability means that explanations are more stable and reliable. This is tested by identifying common features through running trials under similar settings. Based on such a stability test, an XAI method with higher stability can be trusted more by the safety drivers in the anomaly detection process when testing AVs.
**(4)**  **Efficiency:** The efficiency of an XAI method refers to the time it takes to produce an explanation. This metric is crucial as it gauges the XAI method’s suitability for real-world applications, where quick generation of explanations is preferred for practicality. Given that the primary aim is to assist security analysts, the ideal is to provide accurate XAI explanations in real time for timely intrusion detection, particularly in the safety-critical application of autonomous driving systems.
**(5)**  **Robustness:** This metric validates the invariance of explanations to minor perturbations in the input features. Robust techniques should exhibit insensitivity to inconsequential noise or distortions in the data. The robustness of an XAI method refers to its ability to provide consistent explanations even when there are small changes in the data. These changes could be due to errors or intentional attacks. In our study, we used an adversarial model inspired by previous research [22]. This model involves training one biased model that relies heavily on a single feature and another model with all features, including a new one engineered to deceive the XAI explanation method. By generating explanations for normal samples that seem convincing but are actually misleading, there is a risk of compromising the framework’s integrity. This could lead to misidentifying an anomalous AV as a normal one, as the explanations may not accurately reflect the underlying behavior of the autonomous vehicle.
**(6)**  **Completeness:** This metric assesses the capacity of an XAI technique to provide valid explanations for all possible model inputs, including corner cases. More complete methods leave less opportunity for adversaries to exploit blind spots. The completeness of an XAI method means it can provide accurate explanations for all types of samples, even uncommon ones. It is important to note that a complete XAI method is also more robust, meaning it is better at detecting whether an explanation is valid. In our study, we measure completeness by ensuring that every sample has a valid explanation, while the robustness metric focuses on how well the XAI framework resists adversarial attacks.

We analyze these aforementioned six evaluation measures for two popular black-box XAI algorithms on two autonomous driving datasets. The first dataset is the VeReMi dataset [23], which was introduced for anomaly detection in autonomous driving and considers different types of attacks, including denial-of-service (DoS) attacks, Sybil attacks, and message falsification in VANETs. The second dataset is the Sensor dataset [24], which has two classes (normal and anomalous) and contains ten features that can exist within each AV. The two XAI approaches are LIME [20] and SHAP [25]. LIME is a local explanation method that uses linear approximation and perturbations to explain how a decision-making model performs on a sample. In contrast, SHAP has both local and global scopes. This method uses the Shapley value idea from game theory to assess the significance of each feature by calculating its associated value. If the AI model performs similarly without that feature, it is considered less important.

Our extensive evaluation shows different important insights about black-box AI methods for anomaly detection in autonomous driving. First, it shows that SHAP outperforms LIME in terms of global explainability and descriptive accuracy on the VeReMi dataset. In particular, SHAP shows a greater drop in AI models’ accuracy when removing top features compared to LIME for the VeReMi dataset. On the other hand, for the Sensor dataset LIME performs better. For the sparsity metric, SHAP also demonstrates superior performance to LIME on both datasets, with a more exponential growth slope indicating that explanations are concentrated in fewer top features. Furthermore, the evaluation shows that SHAP exhibits better stability than LIME (both globally across many samples and locally on individual samples) for both datasets. Moreover, the evaluation shows that LIME is more efficient than SHAP in terms of runtime for almost all models overall except for some minor exceptions. For robustness, our evaluation shows that SHAP and LIME both are vulnerable to adversarial attacks that aim to provide false explanations. However, they still show biased features, giving analysts a chance to detect attacks. For completeness, the evaluation shows that neither SHAP nor LIME achieved full completeness in explaining all samples. However, it shows that LIME is more complete locally while SHAP is more complete on anomalous samples globally.

This study aims to bridge the divide in utilizing black-box XAI methods for anomaly detection in autonomous driving by implementing evaluation and comparison metrics for these XAI techniques. These metrics encompass security criteria such as completeness, robustness, and efficiency, as well as general AI model attributes like descriptive accuracy, sparsity, and stability. Therefore, our proposed holistic framework aids in selecting optimal XAI techniques for robust and explainable anomaly detection in the security-critical autonomous driving domain.

**Summary of contributions:** The main contributions of this paper can be summarized as follows:

- We introduce a comprehensive framework to assess XAI techniques for anomaly detection in autonomous vehicles. This framework allows for the examination of both global and local XAI methods to gain insights into the decision-making processes of AI models that identify unusual behavior in autonomous vehicles.
- We scrutinize six distinct evaluation metrics for two widely used black-box XAI techniques: SHAP and LIME.
- We validate our XAI evaluation framework via using two prominent autonomous driving datasets (VeReMi and Sensor) across six different AI models.
- We make our source codes publicly accessible, encouraging their use as a foundational XAI evaluation framework for anomaly detection in autonomous driving. Researchers are invited to build upon and create additional models based on this resource (the URL for our source codes of the framework is https://github.com/Nazat28/EXAI_ADS (accessed on 25 May 2024)).

**Paper organization:** The structure of the rest of the paper is as follows: Section 2 covers the related literature. Section 3 outlines the background and problem statement. Section 4 introduces the primary components of our XAI evaluation framework. Section 5 discusses the evaluation of our framework based on six XAI evaluation metrics. Limitations and discussion are addressed in Section 6. Finally, Section 7 offers concluding remarks and outlines future research directions.

## 2. Related Works

**Anomaly detection in autonomous driving:** Several studies have been performed on the anomaly detection side in the autonomous driving field [10,26,27]. In Ref. [26], a modified convolutional neural network (M-CNN) was used on sensor data to detect anomalies in an AV. Ref. [10] utilized CNN and the Kalman filter to detect abnormal behaviors in AVs. The authors in [27] utilized long short-term memory (LSTM) deep networks to determine false data injection (FDI) attacks to ensure stable operation of AVs. However, our work is more focused on anomaly detection from the perspective of XAI evaluation and feature understanding. There are several works that considered anomaly detection for networks of vehicles [9,28,29]. In [30], a hybrid deep anomaly detection (HDAD) framework was employed, enabling autonomous vehicles (AVs) to detect malicious behavior using shared sensor network data. Additionally, the approach in [28] utilized time-series anomaly detection to identify cyber-attacks or sensor malfunctions. Previous research in [9] applied a CNN-based LSTM to differentiate signals from various sources as either anomalous or normal in AVs. In contrast, our framework introduces a systematic approach to evaluate the XAI methods employed to identify key features of an AV and determine the reasons for classifying an AV as either benign or anomalous.

**AI model explanation using XAI:** There are a few studies that have considered applying XAI for anomaly detection for autonomous driving [12,31]. In [12], the authors explained the anomaly detection of AVs with the help of decision tree, random forest, and AdaBoost models but did not interpret such models. Ref. [31] used XAI for all features to identify which features contributed to anomalies in an industrial control system (ICS). In our study, we explain the contribution of each feature with an anomaly score in two different datasets (VeReMi and Sensor) using six different AI models in a vehicle-to-vehicle (V2V) network. In [32], the authors reviewed different classifier models for anomaly detection in intelligent transportation systems and suggested the need for XAI to interpret them. Ref. [33] utilized XAI to represent the behavior of an in-vehicle intrusion detection system (IV-IDS) in a DNN for detecting anomalies to thwart cyber-attacks in vehicles. On the other hand, in our work, we use XAI to interpret the classification decisions of six black-box AI models using two datasets. Moreover, we implement two novel feature selection methods to extract the main features.

**Feature extraction for securing AVs:** Feature extraction reduces the redundant data and improves the computation time and the prediction accuracy of the AI model (on the testing data) [34]. Extraction of important features can also help to secure an AV. For example, feature extraction can be helpful to detect anomalous behavior of an AV or identify prospective intruders that may reside in the VANET by analyzing the data’s patterns [1]. Feature extraction has been previously utilized in securing the CAN bus in AVs [35], where a new feature called the “time interval” was introduced to improve the performance of AI models. In [36], the authors reduced the number of features based on importance in detecting intrusion using a back propagation neural network. To find the confidential real-time position of an AV. Ref. [37], introduced a new attack on an AV to show the vulnerabilities of the AV. Moreover, ref. [38] proposed a feature extraction technique on encrypted data in order to prevent private information leakage to secure the AV from a communication standpoint. In our framework, we propose a novel method for evaluating XAI-based feature selection techniques and implement it through different black-box AI models on two datasets to detect anomalies in AVs.

**XAI for feature selection in autonomous driving:** While anomaly detection and XAI methods have been explored for autonomous vehicles, there is limited prior work leveraging XAI techniques for feature selection in this domain. Recent studies have begun investigating the use of XAI for identifying influential features in autonomous systems. Ref. [39] applied SHAP to select key lidar features for improving uncertainty estimates in 3D vehicle detection models for autonomous driving. Pruning low-SHAP-value features reduced model uncertainty without sacrificing performance. Ref. [40] employed RNNs with SHAP-based feature selection for imputing missing values and feature evaluation in multivariate autonomous driving time-series data. Overall, these works highlight the promise of XAI-guided feature selection for improving efficiency, uncertainty characterization, and real-time explainability in autonomous driving systems. However, in our work, we systematically assess XAI methodologies against predefined criteria, aiming to ascertain their efficacy in engendering trust for practical deployment within real-world contexts.

**XAI evaluation:** While there are a couple of studies [21,41] that have utilized the six metrics we mentioned to assess XAI methods, our work has distinct differences in scope. Firstly, the application domain of these prior studies [21,41] focused on network intrusion detection within computer networks. In contrast, our paper introduces an end-to-end framework tailored for evaluating XAI techniques in the context of anomaly detection in autonomous vehicles. Here, our aim is to evaluate XAI methods that elucidate the decision-making process of AI models classifying anomalous AV behavior.

Secondly, the nature of the datasets used in our work diverges from those in the aforementioned studies. The datasets in the prior research [41] predominantly consist of network traffic data (refer to Tables 1 and 2 in [41]). In our study, however, we primarily utilize two popular autonomous driving datasets, namely, VeReMi and Sensor. These datasets comprise data collected from sensors and message logs of the autonomous vehicles’ on-board units (OBUs). Specifically, the VeReMi dataset, generated from a simulation environment, provides message logs from OBUs along with labeled ground truth. Furthermore, the Sensor dataset includes data from the primary communication sensors of an autonomous vehicle.

These two distinctions lead to notable differences in the emphasis of each metric and the primary findings related to some of our six evaluation metrics.

## 3. The Problem Statement

We now provide the main preliminaries for anomaly detection in autonomous driving, the challenges of black-box AI, and the need for XAI methods and the accompanying challenges in their evaluation.

### 3.1. Securing Autonomous Driving Systems

Ensuring the safety and security of autonomous driving systems is paramount, requiring careful measures to safeguard them [42]. Robust security practices include implementing strong authentication mechanisms, regular software updates, employing multilayered defense strategies, conducting risk assessments, and adhering to regulatory compliance. Additionally, continuous surveillance and thorough testing are crucial for identifying and rectifying security vulnerabilities. By prioritizing and effectively implementing these security measures, the risk of security incidents and potential harm to passengers and other road users can be significantly minimized. To achieve these goals, it is advisable to deploy meticulously structured AI models on autonomous driving datasets that encompass a mix of benign and anomalous data. Such models can effectively differentiate between benign and anomalous autonomous vehicles (AVs). Successful classification can aid law enforcement in locating and apprehending anomalous AVs, thereby contributing to the overall safety of autonomous driving systems.

### 3.2. Shortcomings of Black-Box AI Models

While AI models have played a crucial role in enhancing the security of autonomous driving systems, their inherent black-box nature poses challenges in understanding the intricate relationships among features within these models. The complexity of these models makes it difficult to comprehend how and why they achieve specific results. This black-box problem is a common issue encountered by many AI models. The literature underscores the black-box nature of AI models and the need for interpretability [43,44]. Even though these AI models have demonstrated effective deployment in various autonomous driving sectors with high prediction accuracy, explaining their behavior, especially in cases of errors, remains challenging. To address this issue in the safety-critical domain of autonomous driving, proposing XAI frameworks that elucidate the decision-making process of AI models is essential.

### 3.3. Explainable AI

Given the inherent black-box nature of AI models, there is a pressing need for a strategy that comprehends and interprets their operations [45]. This necessity has driven the development of XAI methods. XAI interfaces visualize the outputs of various data points, providing insights into the relationships between specific features and model predictions [46]. XAI is proficient in explaining the behavior of AI models both globally and locally. Notable methods in XAI include Shapley additive explanations (SHAP) and local interpretable model-agnostic explanations (LIME). SHAP primarily relies on the Shapley value concept from game theory [19]. On the other hand, LIME aids in explaining an AI model based on single instances via creating a local model that approximates the behavior of the global AI model. We focus on evaluating these two XAI methods.

### 3.4. Benefits of Applying Explainable AI for Securing Autonomous Vehicles

Applying XAI to enhance the security of autonomous vehicles (AVs) offers several advantages. Firstly, XAI facilitates the identification of safety risks and the implementation of practical solutions by providing a comprehensive understanding of how autonomous driving systems make decisions. Secondly, XAI makes black-box AI models interpretable to humans. For instance, if the autonomous driving system employs sensors and cameras to detect pedestrians and obstacles, an XAI algorithm can offer clear explanations of how the system decides on evasive actions, such as braking or swerving, upon detecting a pedestrian. Research such as Ref. [47] underscores that systems delivering understandable explanations significantly gain user trust. Additionally, Ref. [48] emphasizes the need for algorithmic explanations to build trust in autonomous systems. XAI provides users and regulators with more information and insights, enhancing the accuracy of autonomous driving. Furthermore, XAI offers appropriate explanations regarding the classification of AVs. Thus, XAI can play a crucial role in ensuring that these autonomous driving systems are accountable for their actions, aiding in post-accident investigations [49].

### 3.5. Challenges of XAI for Securing Autonomous Vehicles and Need for Evaluating XAI

Applying XAI to anomaly detection in autonomous driving presents several challenges. Primarily, no black-box XAI technique can produce explanations that meet all six evaluation metrics flawlessly, as demonstrated in our results in Section 5 and in the study that established these metrics [21]. The existing challenges can be classified as follows:

- **Transparency challenges:** Anomaly detection AI models for intrusion often operate as black boxes, posing significant difficulties for interpretation and understanding. This lack of transparency is especially problematic for autonomous driving security, restricting the ability of security experts and safety drivers to effectively audit and protect these systems against potential threats.
- **Limited application of XAI in autonomous driving security:** The unique challenges posed by anomaly detection in autonomous driving [50] have led to a limited development of interpretative methods for anomaly detection systems in this domain. This contrasts with other fields, like text analysis and computer vision, where a broader range of XAI methods has been established. This limitation restricts the effective use of XAI in enhancing autonomous driving security.
- **Accuracy and robustness:** In addition to accuracy, XAI methods utilized in autonomous driving security systems must fulfill additional criteria, including delivering comprehensive and resilient explanations. The provision of complete and robust explanations enhances the reliability of XAI methods, ensuring their effective application in the safety-critical autonomous driving security application.
- **Evaluation and comparison:** There is an urgent requirement to establish evaluation criteria for assessing XAI methods and facilitating comparisons within the anomaly detection domain in autonomous driving [51]. These criteria should encompass the specific demands of the autonomous driving field, including stability, robustness, reliability, and efficiency, along with general properties inherent in machine learning models such as accuracy, transparency, and explainability.

Having provided the problem statement and the main challenges of using XAI for securing autonomous vehicles, we next detail our proposed XAI evaluation framework.

## 4. Framework

### 4.1. An End-to-End XAI Pipeline for Autonomous Driving Systems

**Figure 1:**
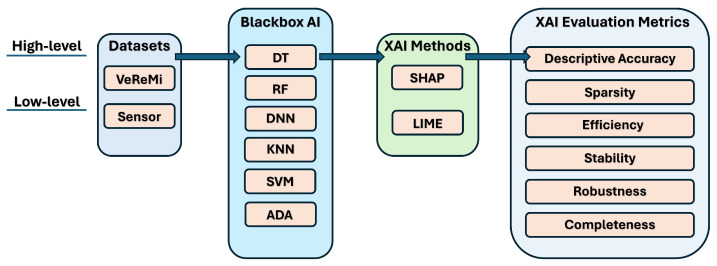
An overview of our XAI evaluation framework for anomaly detection in autonomous driving systems. The framework assesses six different metrics for two popular XAI methods.

The principal objective of this research is to construct an XAI evaluation pipeline that derives salient quantitative metrics to systematically analyze the performance of prevalent XAI techniques (such as SHAP and LIME) on autonomous driving data. The pipeline will facilitate the selection of optimal XAI methods that can better elucidate model predictions for developers and safety analysts in autonomous driving applications. To achieve this goal, we propose an end-to-end XAI evaluation pipeline. The different components of our pipeline (shown in Figure 1) are explained below.

**High-level XAI evaluation pipeline:** At a broad level, the pipeline ingests raw autonomous driving datasets encompassing sensor, position, and speed data of an AV. These are the main inputs into multiple black-box AI models to generate predictions (whether an AV has anomalous behavior or not). Subsequently, the predictions are analyzed by XAI techniques (including SHAP and LIME) to produce model’s top features and explanations. These explanations are then evaluated on the following six key dimensions: descriptive accuracy, sparsity, stability, efficiency, robustness, and completeness.

**Low-level XAI evaluation pipeline:** We now explain the low-level components of our XAI pipeline. The different components (shown in Figure 1) are detailed below:

**(i)** 
**Loading autonomous driving dataset:** In this study, we used two different datasets. One is the vehicular reference misbehavior (VeReMi) dataset, which is a dataset to analyze misbehavior detection mechanism in VANETs. Generated from simulation environment, this dataset provides message logs of on-board units (OBUs) and labeled ground truth [23]. The other one is a dataset created based on the Sensor dataset [2], that contains data from the main communication sensors of an AV. We want to underline that when choosing the Sensor data for each AV in this dataset, we adhered to the data ranges provided by prior works [24,52].
**(ii)** 
**Black-box AI models:** After completing the dataset preprocessing, we proceed to train the black-box AI models. We split the data, allocating 70% for training and reserving the remaining 30% for testing. We develop six models: decision tree (DT), random forest (RF), deep neural network (DNN), k-nearest neighbors (KNN), support vector machine (SVM), and AdaBoost (ADA). The hyperparameters for these AI models were fine-tuned to achieve optimal predictive performance (refer to Appendix A).
**(iii)** 
**XAI methods:** We explain the black-box XAI methods using SHAP and LIME as prototypical examples of model-agnostic post hoc explanation methods suitable for diverse AI models. Post-construction of the black-box AI models, we leverage two prevalent model-agnostic post hoc XAI techniques—LIME [20] and SHAP [25]—to elucidate the models. These encompass both global and local scopes for comprehensive analysis. As model-agnostic approaches applied after model training, they are compatible with diverse AI models. Specifically, SHAP derives explanations by attributing an importance score to features based on Shapley values from game theory. In contrast, LIME approximates the global model locally using linear surrogate models to explain individual predictions. Although designed for a local scope, we modified LIME to generate global explanations by aggregating local feature importance scores over many samples. For each sample, LIME produces local explanations with feature scores. We then accumulate the scores for each feature across samples, and then, average the summed absolute score values per feature across samples. Finally, we rank these features by average importance and select the top features as globally influential. This allows LIME to provide global insights without re-engineering.
**(iv)** 
**XAI evaluation metrics:** We assess the XAI methods using six key metrics: descriptive accuracy, sparsity, stability, efficiency, robustness, and completeness. Descriptive accuracy gauges the reduction in AI model accuracy when the top influential features, identified by XAI methods, are omitted. Sparsity assesses whether the explanations are centralized on a few key features or spread across numerous less significant ones. Stability examines if the explanations vary significantly across multiple runs. Efficiency evaluates the time each method requires to produce explanations, favoring quicker methods. Robustness determines whether explanations can be deceived by adversarial input changes intended to misguide the XAI method. Lastly, completeness examines whether explanations offer a thorough understanding of the AI model’s decision making, even for outlier samples.

#### Step-by-Step Process for Producing XAI Evaluation Metrics

To produce our XAI evaluation measures and their outcomes, we next provide the detailed procedure for each of our six evaluation metrics (descriptive accuracy, sparsity, efficiency, stability, completeness, and robustness).

Algorithm 1 shows the algorithm of the descriptive accuracy metric. The main idea is to systematically remove the most important features and see how this affects the accuracy of different AI models on the two datasets. We test this metric for each AI model.
**Algorithm 1** Descriptive Accuracy Metric Algorithm**Require:** 
VeReMi dataset, Sensor dataset, List of AI models (DT, RF, DNN, KNN, SVM, ADA), Set of number of top-*k* features where k={0,2,4,6,8}**Ensure:** 
Descriptive Accuracy graphs for each AI model  1:**for** each AI model **do**  2:    **for** each dataset **do**  3:        features = get_ranked_features(dataset, AI_model);  4:        **for** $k_{i}\in k$ **do**  5:           reduced_features = remove_top_*k*_features(features, $k_{i}$);  6:           accuracy = train_and_evaluate(AI_model, dataset, reduced_features);  7:           plot_point($k_{i}$, accuracy);  8:        **end for**  9:        create_descriptive_accuracy_graph();10:    **end for**11:**end for**12:**return** Descriptive Accuracy

Algorithm 2 shows the algorithm of the sparsity metric. The idea is to understand how the sparsity of feature scores (i.e., the proportion of features with scores below a certain threshold) changes as the threshold varies, for different AI models and datasets. The AUC provides a summary measure of the sparsity across all thresholds.

Algorithm 3 shows the algorithm for the stability metric. The idea is to assess how consistent the explanations provided by the XAI method are across multiple runs on the same dataset. A higher stability score (closer to 1) indicates that the explanations are more reliable and reproducible, while a lower score (closer to 0) suggests that the explanations may be sensitive to random variations or other factors, leading to inconsistent explanations.
**Algorithm 2** Sparsity Metric Algorithm**Require:** 
VeReMi dataset, Sensor dataset, List of AI models (DT, RF, DNN, KNN, SVM, ADA)**Ensure:** 
Sparsity graphs and AUC for each AI model  1:Obtain feature scores using XAI techniques like SHAP or LIME.  2:Normalize the feature scores to be between 0 and 1 using a min-max technique.  3:**for** each AI model **do**  4:    **for** each dataset **do**  5:        Create an X-axis with values ranging from 0.0 to 1.0 in steps of 0.1 (0.0, 0.1, 0.2, 0.3, 0.4, 0.5, 0.6, 0.7, 0.8, 0.9, 1.0). These values represent the threshold that we compare with the feature scores.  6:        **for** each threshold value τ on the X-axis **do**  7:           Calculate the sparsity score using the following formula:
$$\begin{array}{cc} \mathrm{Sparsity} & =\frac{\mathrm{Number}\mathrm{of}\mathrm{Features}s.t.\mathrm{Feature}\mathrm{Score}\leq \tau }{\mathrm{Total}\mathrm{Number}\mathrm{of}\mathrm{Features}} \end{array}$$  8:           Plot the point (τ,Sparsity) on the graph.  9:        **end for**10:        Calculate the area under the curve (AUC) from the plot obtained.11:    **end for**12:**end for**13:**return** Sparsity, AUC

**Algorithm 3** Stability Metric Algorithm
**Require:** 
VeReMi dataset, Sensor dataset, List of AI models (DT, RF, DNN, KNN, SVM, ADA)**Ensure:** 
Stability scores for each AI model  1:**for** each AI model **do**  2:    **for** each dataset **do**  3:        Choose the top-*k* features to compare across multiple runs of the XAI method. The value of *k* depends on the total number of features in the dataset.  4:        Check the stability of the XAI method across multiple runs using this formula:
$$\begin{array}{cc} IS(i,j) & =\frac{|T_{i}\cap T_{j}|}{k} \end{array}$$
where IS(i,j) is the stability score, which represents the ratio of common top features across two runs *i* and *j*. $T_{i}$ and $T_{j}$ are the sets of top-*k* features identified by the XAI method in runs *i* and *j*, respectively. $|T_{i}\cap T_{j}|$ is the number of common features between the two sets $T_{i}$ and $T_{j}$. *k* is the total number of top features.  5:        If all the top-*k* feature sets are the same across different runs, the stability score will be 1, indicating perfect stability.  6:        If none of the top features are the same across runs, the stability score will be 0, indicating that the XAI technique is not stable and produces different explanations in each run.  7:    **end for**  8:
**end for**
  9:**return** Stability Score (*IS*(*i*,*j*))


Algorithm 4 shows the algorithm for the efficiency metric. The idea is to understand the computational efficiency of different XAI methods (SHAP and LIME) when applied to various AI models and datasets, and how this efficiency changes as the number of samples increases. By measuring the time taken for the XAI evaluation process, we can identify the most efficient combinations of XAI method and AI model for different scenarios, which can be useful for real-world applications where computational resources may be limited.
**Algorithm 4** Efficiency Metric Algorithm**Require:** 
VeReMi dataset, Sensor dataset, List of different AI models**Ensure:** 
Efficiency metrics for each AI model  1:**for** each AI model **do**  2:    **for** each dataset **do**  3:        After training a given AI model, apply LIME or SHAP to explain the model’s predictions for different numbers of samples.  4:        **for** each combination of AI model, XAI method, dataset, and number of samples **do**  5:              Measure the time it takes to complete the XAI evaluation process.  6:        **end for**  7:        Summarize the results and analyze which combination of XAI technique and AI model is the most efficient for different numbers of samples.  8:    **end for**  9:**end for**10:**return** Efficiency Metrics (Runtime of XAI for different AI models)

Algorithm 5 shows the main algorithm for the robustness metric. The idea is to evaluate the robustness of XAI explanations by intentionally introducing biases and unrelated features, and checking if the XAI methods can correctly identify these factors in their explanations. The occurrence and sensitivity graphs help visualize and analyze the robustness of the XAI methods under adversarial conditions.
**Algorithm 5** Robustness Metric Algorithm**Require:** 
Dataset, Original model, List of XAI methods (SHAP, LIME)**Ensure:** 
Robustness and Sensitivity graphs  1:Train an adversarial model and a biased model to decouple the original model’s predictions from the XAI explanations.  2:**for** each model type (adversarial, biased) **do**  3:    **if** adversarial model **then**  4:        Use the top-k anomalous features as unrelated features.  5:    **else if** biased model **then**  6:        Use a single biased feature.  7:    **end if**  8:    **for** each XAI method (SHAP, LIME) **do**  9:        Check if the biased feature appears as the top feature for the biased model.10:        Check if the unrelated and biased features appear in the top 3 features for the adversarial model.11:    **end for**12:**end for**13:Generate an occurrence graph by running the Robustness metric for 1000 samples. Record the top 3 features for each model in a bar graph.14:Analyze the occurrence of each feature for each model. The biased model should have the biased feature as the top feature.15:For the adversarial model, verify if the unrelated and biased features are among the top 3. If the unrelated feature ranks higher than the biased feature, the explanation was hijacked.16:Generate a sensitivity graph as per [21]:17:**for** each OOD classifier model **do**18:    Train the model and extract its F1 score (X-axis).19:    Run the XAI explanation for the adversarial models.20:    Count the number of times the biased feature appears as the top feature (Y-axis).21:**end for**22:**return** Robustness and Sensitivity Metrics

Finally, we evaluate the completeness of the XAI explanations by perturbing (modifying) the top features for a given sample and checking if the predicted class changes. The percentage of samples whose predictions remain valid (unchanged) after perturbations quantifies the completeness of the explanations. We follow the approach from Ref. [53], where our algorithm is shown below.

Algorithm 6 shows the main algorithm for the completeness metric. The idea is to systematically perturb the most important features identified by the XAI explanation and see if the model’s prediction remains consistent. If the prediction changes after perturbing a feature, it suggests that the XAI explanation is incomplete and may be missing important factors that influence the model’s decision. A higher completeness score (closer to 100%) indicates that the XAI explanations are more complete and capture the essential factors contributing to the model’s predictions.
**Algorithm 6** Completeness Metric Algorithm**Require:** 
Dataset, Original model, List of XAI methods**Ensure:** 
Completeness score  1:Group the samples based on their anomaly label classes.  2:Generate XAI explanations for the original, unperturbed samples.  3:**for** each sample **do**  4:    **for** each top-k feature **do**  5:        Modify the top-ranked feature and check if the anomaly prediction class changes.  6:        Adjust the feature’s value from 0 to 1 in increments of 0.1 during perturbation.  7:        **if** changing the current perturbed feature doesn’t alter the anomaly prediction class **then**  8:           Set its value to the opposite of the original value to maintain the relevance of previous perturbed features.  9:        **else**10:           Stop the experiment for that sample and record the change.11:        **end if**12:    **end for**13:**end for**14:Count the number of samples whose prediction class changed during perturbation.15:Divide this by the total number of samples in the batch.16:Calculate the percentage of samples whose predictions remained valid (unchanged) after perturbations. This percentage is the final completeness score.17:**return** Completeness Scores

### 4.2. Top Features List in the Datasets

We now present the whole list of characteristics used in constructing AI models, as well as their explanations, for the VeReMi dataset and the Sensor dataset, as utilized in our framework. Table 1 and Table 2 describe each feature in the Sensor and VeReMi datasets, respectively.

## 5. Evaluation

Next, we present our comprehensive evaluation results. Our assessment seeks to address the following research questions:

- How can XAI elucidate the decision-making processes of AI models in identifying anomalous autonomous vehicles?
- How do the two XAI techniques perform across the six evaluation metrics?
- What are the advantages and drawbacks of employing black-box XAI methods for anomaly detection in autonomous driving?
- Which black-box XAI method performs better across the six evaluation metrics?

### 5.1. Dataset Description

**VeReMi dataset** [23]**:** This dataset was introduced for anomaly detection in autonomous driving. It considers different types of attacks, including denial-of-service (DoS) attacks, Sybil attacks, and message falsification in VANETs. Each of these attack scenarios has associated real-world data (both readings and ground truth labels). The VeReMi dataset includes AV message logs and an attacker’s ground truth file to identify the characteristics of the attacker. It consists of several individual scenarios and five different attacker types [23]. This dataset is considered as a benchmark in the field of security of autonomous driving [23]. In our framework, we formulated the problem as a binary classification task, categorizing network traffic as either anomalous or benign. Specifically, we labeled the five attack types under consideration as anomalous samples, while all other samples were treated as benign for purposes of evaluation and analysis of the six XAI evaluation metrics (except for the efficiency metric). For selecting the features of the VeReMi dataset, we extracted the features that describe the behavior of an AV. First, we reduced the columns to only pos_x, pos_y, pos_z, spd_x, spd_y, and spd_z, as the other columns did not provide much information. Features pos_x, pos_y, pos_z and spd_x, spd_y, and spd_z represent the position of an AV in the x, y, z directions and speed in the x, y, z directions, respectively.

**Sensor dataset** [24]**:** We also employed the Sensor dataset to assess our framework, adhering to the methodology outlined in [24]. This dataset encompasses ten features, which we presume are present in each autonomous vehicle (AV). These features include formality, location, speed, frequency, correlation, lane alignment, headway time, protocol (message sequence), plausibility, and consistency sensors. The formality sensor verifies the correct message size and header. The location sensor confirms if the message reaches its intended destination. The speed sensor ensures the data range remains within the speed limit. The frequency sensor examines the timing behavior of the messages. The correlation sensor determines if various messages comply with the defined specifications. The lane alignment sensor assesses if the AV remains within its lane. The headway time sensor checks if the headway distance is maintained. The protocol sensor verifies the correct sequencing of messages. The plausibility sensor gauges the relative difference in size between consecutive messages. The consistency sensor ensures data from different sources align consistently. A detailed description of each sensor (feature) is provided in Table 1. These sensors serve to identify the operational mode (normal or malicious) of each AV. The normal data range for the Sensor dataset was specified. Thus, any AV that deviates from this normal range can be classified as anomalous.

**Summary and statistics of the datasets:** Table 3 shows the size, number of samples, and attack types of the two datasets.

### 5.2. Experimental Setup

**Coding tools:** We used several open-source tools (based on Python) and various black-box AI models using well-known libraries like Keras [54] and Scikit-learn [55] in order to create our framework. Also, the following XAI toolboxes were used:

**(a)** 
**SHAP** [56]: The predictions of AI models are explained by SHAP. It was created based on a game theory concept (Shapley value) and can evaluate the contributions of each characteristic (feature) to the classification of any AI model.
**(b)** 
**LIME** [57]: It is a form of XAI model that aids in explaining an AI model locally and making each prediction understandable on its own. This approach describes the features that contributed to the decision of the classifier on a single instance.

**AI models:** To evaluate our framework, we paired six different types of AI models (deep neural network (DNN) [58], random forest (RF) [59], AdaBoost (ADA) [60], k-nearest neighbor (KNN) [61], support vector machine (SVM) [62], and decision tree (DT) [45]) to explain the black-box characteristics of these models on two datasets using our XAI methods. We present the main hyperparameters we used for each AI model in Appendix A.

### 5.3. Evaluation Metrics

**Metrics for XAI:** To explain the characteristics of the considered AI models, we generate the following XAI metrics: global summary plots using SHAP (for overall feature rank), local explanation by LIME and SHAP (to explain the contribution of each feature to the classification for a single data instance), and feature ranking (to explain the main features that identify anomalies).

**Metrics for XAI evaluation:** To assess XAI techniques, we employ the following metrics:

(1) Descriptive accuracy: This metric is presented through accuracy (ACC) figures for each AI model and XAI technique across datasets (refer to Figures 4 and 5).
(2) Sparsity: We use tables to display the area under the curve (AUC) for each model and XAI technique per dataset. This offers a unique perspective on explainability (see Table 4 and Table 5). Notably, a lower AUC is preferable for descriptive accuracy, whereas the opposite is true for sparsity.
(3) Efficiency: The efficiency tables for each XAI technique indicate the time taken to produce XAI explanations for both local (single instance) and global (multiple instances) scopes (see Tables 10–12).
(4) Stability: A stability table is crafted to evaluate the reliability of XAI explanations. This experiment repeatedly generates top features for each XAI method and examines the intersection of these features across different trials under identical conditions (refer to Tables 6–9).
(5) Robustness: Drawing from the code in [22], we created an adversarial model that disconnects the sample from its explanation. We tested the ability to produce false predictions while still presenting plausible explanations. For example, attempting to classify an anomalous AV as normal while offering a convincing XAI rationale for such a decision (see Figures 9 and 10).
(6) Completeness: We assess whether XAI techniques can achieve full completeness, covering genuine explanations for all network traffic instances, including edge cases. For instance, if a perturbation alters the explanation of the top features without changing the predicted class, such an explanation cannot be trusted. Therefore, our completeness figures and tables (see Tables 13 and 14 and Figures 20 and 21) gauge whether the most influential features modify the outcomes when sufficiently perturbed.

**Table 4:** Quantitative results for sparsity metric for VeReMi dataset. We show the area under the curve (AUC) for different AI models for both XAI methods (SHAP and LIME). We emphasize that SVM shows the best sparsity for SHAP.

XAI Methods	DT	RF	DNN	KNN	SVM	ADA
SHAP	0.68	0.71	0.78	0.63	0.95	0.71
LIME	0.63	0.58	0.65	0.60	0.56	0.63

**Table 5:** Quantitative results for sparsity metric for Sensor dataset. We show the area under the curve (AUC) for different AI models for both XAI methods (SHAP and LIME). We emphasize that DT gives the best sparsity for SHAP while SVM shows the best sparsity for LIME.

XAI Methods	DT	RF	DNN	KNN	SVM	ADA
SHAP	0.76	0.62	0.73	0.67	0.57	0.71
LIME	0.62	0.62	0.53	0.65	0.70	0.59

After outlining the primary experimental setup, we proceed to present detailed evaluation results using our two autonomous driving datasets.

### 5.4. Evaluation Results

#### 5.4.1. The Importance of Features via Explainable AI

We now show the importance of top features that affect the decision of each AI model we consider for the two datasets.

**Figure 2:**
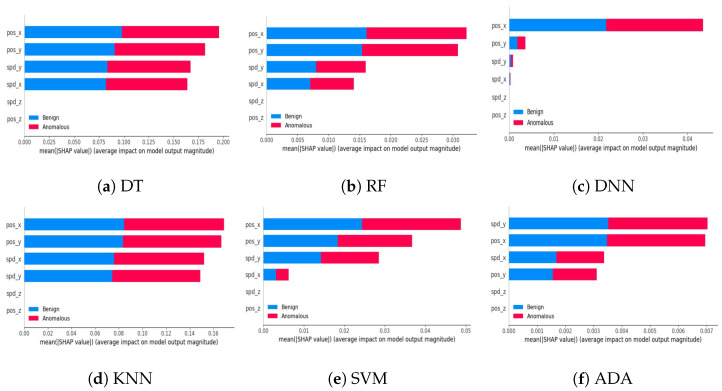
Feature importance using global summary plot (generated by SHAP) for each classifier AI anomaly classification model for the VeReMi dataset. It also shows the importance level of these features for each classification type (different colors). We observe that pos_x is the most important feature for the VeReMi dataset for all AI models except ADA.

**Figure 3:**
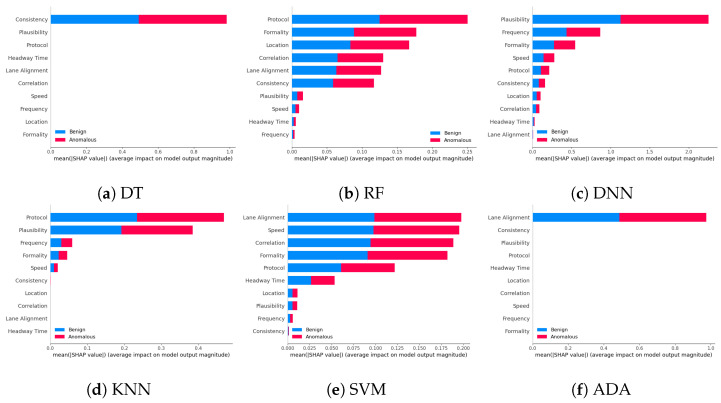
Feature importance using global summary plot (generated by SHAP) for each AI anomaly classification model for the Sensor dataset. It also shows the importance level of these features for each classification type (different colors). We observe that “lane alignment” and “protocol” are the most frequent top features across AI models for the Sensor dataset.

**Global summary plot:** We initially present global summary plots for each AI model, illustrating the crucial features influencing the model’s decision. These plots display feature importance in a descending order, indicating the most to the least significant features. Figure 2 and Figure 3 showcase feature importance for various AI models applied to the VeReMi and Sensor datasets, respectively. The feature importance values are obtained by averaging the means of all Shapley values. Notably, these figures highlight pos_x and lane alignment as the most influential features for the VeReMi and Sensor datasets, respectively. It is important to note that these figures delineate the significance of different features for each of the two classes of autonomous vehicles (benign and anomalous) in vehicular ad hoc networks (VANETs). By calculating the important features and visualizing their contribution via XAI, an invigilator will be able to identify which features to look at to make a decision on the classification of an AV.

#### 5.4.2. Descriptive Accuracy

We begin the six evaluation metrics by evaluating the level of descriptive accuracy exhibited by XAI techniques on our VeReMi and sensor datasets for autonomous driving. Recall that descriptive accuracy refers to the process of quantifying the significance of each feature. If a feature holds significant importance in the model’s prediction, its removal will result in a drop in the model’s accuracy. Thus, descriptive accuracy of an XAI method refers to the drop in the AI model’s prediction performance when the most influential features identified by the XAI method are removed. A greater decrease in accuracy suggests that the features that were deleted have a strong ability to explain the data. Intuitively, we anticipate a decrease in the accuracy of AI models when we eliminate the top four influential features from the VeReMi dataset and top eight influential features from the Sensor dataset. This is because these numbers of features for the two datasets are the maximum number we eliminated in this work for each dataset.

**Figure 4:**
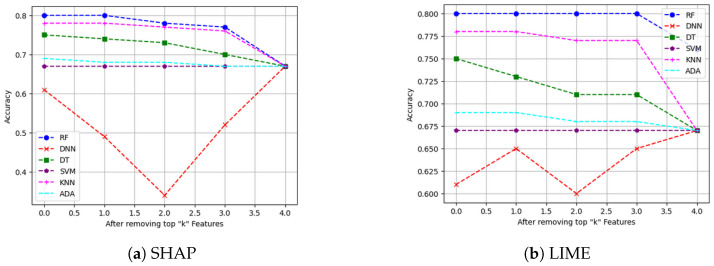
Descriptive accuracy of SHAP and LIME on VeReMi dataset for six different AI models. We remove the top 4 features. We observe that SHAP outperforms LIME in terms of descriptive accuracy. For both SHAP and LIME, the majority of the AI models perform as anticipated, as evidenced by a declining trend, suggesting a decrease in accuracy.

**Figure 5:**
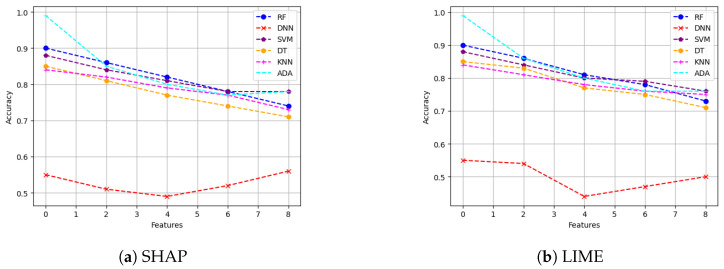
Descriptive accuracy of SHAP and LIME on Sensor dataset for six different AI models. We remove the top 8 features. We observe that the accuracy of all models decreases when the top features are removed from the Sensor dataset except for the DNN for both SHAP (**a**) and LIME (**b**).

**Main insights:** For the VeReMi dataset, according to Figure 4, SHAP outperformed LIME in terms of global explainability. In particular, the majority of the AI models performed as anticipated, as evidenced by a declining trend, suggesting a decrease in accuracy. The optimal scenario would involve a rapid decline in accuracy over time, following an exponential decrease pattern. In our experiment, it is worth noting that not all of the models experienced a decrease in accuracy. The accuracy of the SVM remained constant when the top four features were removed. Furthermore, the accuracy of the DNN exhibited some variability. A potential rationale for this behavior could be the insufficient number of features in our VeReMi dataset. In general, SHAP outperformed LIME in terms of accuracy decline. The decrease in accuracy for SHAP (Figure 4a) was greater compared to LIME, with all models experiencing a drop to approximately 0.68 for SHAP. For LIME (Figure 4b), the accuracy of all models decreases to 0.67, except for RF which decreases to an accuracy of 0.75. For the Sensor dataset, shown in Figure 5, we see that the accuracy of all the black-box AI models decrease when top features are removed from the Sensor dataset, except for the DNN, for both SHAP (Figure 5a) and LIME (Figure 5b). This implies that both SHAP and LIME perform almost the same on this Sensor dataset. Finally, it is worth mentioning that the security analysts should check the decisions of the majority of the models, as in our case DNN does not show the expected descriptive accuracy.

#### 5.4.3. Sparsity of Explanation

**Figure 6:**
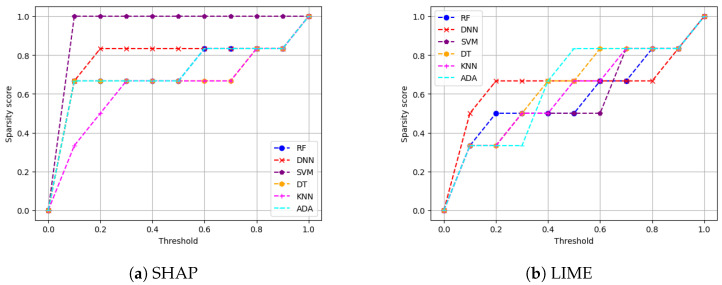
The sparsity metric score of VeReMi dataset for six different AI models for SHAP and LIME. We observe that SHAP shows better performance in comparison to LIME where it has a higher vertical growth in slope compared to that of LIME.

**Figure 7:**
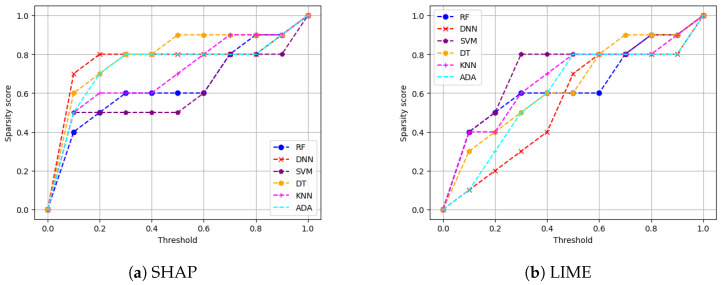
The sparsity metric score of Sensor dataset for six different AI models for SHAP and LIME XAI methods. We see that in the sparsity test both SHAP and LIME perform almost the same in the case of the Sensor dataset.

We then provide the sparsity results of our six models on SHAP and LIME for both of our datsets. Recall that sparsity refers to how many input features are marked as highly relevant by the XAI explanation method. More sparse explanations are desirable as they identify a small subset of important features, making the model easier to interpret. Higher sparsity means more features are discarded as they are irrelevant, and the model is using few features to reach the classification decision. On the other hand, lower sparsity means the model is using more features for reaching the classification decision. For instance, for a given threshold value, if five out of six features are in the range below or equal to that threshold value, this means that one feature is of high importance to the XAI method while the other five are not important. In our experiments, the threshold value was swept in small increments of 0.1 from 0 to 1 in this instance, and the sparsity of each black-box XAI method (here SHAP and LIME) was recorded (as shown in Figure 6 and Figure 7).

**Main insights:** Figure 6 shows the sparsity for both SHAP and LIME for the VeReMi dataset. Again, SHAP shows better performance in comparison with LIME. To be precise, Figure 6a shows a slope with high vertical growth in the left-hand side of the graph than that of LIME (Figure 6b), which means the explanation is concentrated only in a few top features which is the ideal case. Table 4 shows the area under the curve (AUC) for the SHAP and LIME sparsity points. The table shows that for SHAP, the sparsity is higher than that of LIME for all six models considered in this work (i.e., that is why the AUC of every black-box AI model for SHAP is greater than that of LIME). Therefore, for SHAP the security analysts managing autonomous driving systems will have to look at a small portion of features for making decisions as high sparsity means the AI model depends on a small number of features to reach its decision. However, for LIME (Figure 6b) the sparsity is lower, which indicates that LIME has to take more features into account than SHAP to reach its decision (i.e., generating its explanations).

Again for the Sensor dataset, from Figure 7 we can see that in the sparsity test both SHAP and LIME perform almost the same in this case as well. However, Table 5 indicates that SHAP performs a bit better as the area under the curve (AUC) for SHAP is greater for most of the AI models than that of LIME. Lower sparsity is harder for explainability since the analysts will have to look at a large portion of features for making decisions as low sparsity means the model depends on a large number of features to reach its decision.

#### 5.4.4. Stability

We then calculate the stability of the XAI explanations. For this case, we emphasize that we followed the procedure stated in Ref. [21]. First, we run each XAI explanation method on the VeReMi dataset three times. For each run, we store the top three features across the six models. Similarly, for the Sensor dataset, we consider the top five features. We next compute the intersection size between the top feature sets from different runs as shown in Algorithm 3. The intersection size gives a measure of stability on a scale of 0 to 1, where 1 indicates the top features were identical between runs (the XAI method does not change the explanation in different identical runs) and 0 indicates no overlap in top features (explanations change with each run since they have totally different top features).

**Main insights of global stability:** We first conducted a comparison of the average percentage of common top features across runs between the SHAP and LIME approaches for the VeReMi dataset, both on a global scale (using many samples) and on a local scale (using a single sample). The purpose was to identify which method yielded the most consistent explanations throughout multiple runs. In our analysis of global stability (shown in Table 6), SHAP demonstrates superior performance since the average intersection size for SHAP is greater compared to LIME. In the global stability experiment for the Sensor dataset (shown in Table 7), SHAP outperforms LIME in three of the models and has the same stability score as LIME for two of the AI models. However, LIME outperforms SHAP in one model, which is RF. Overall, SHAP performs better in terms of global stability for the Sensor dataset.

**Main insights of local stability:** For the local stability test for the VeReMi dataset (shown in Table 8), SHAP showed higher stability in comparison with LIME on average. We emphasize that we tested each model on the same sample multiple times and listed the top three stable features. For SHAP, more AI models show stability than for LIME (i.e., SHAP has the same or better stability in all six AI models). For the case of the Sensor dataset we stored the top five stable features. Table 9 shows that SHAP outperforms LIME in four of the models in terms of local stability. However, LIME performs better than SHAP in one model (decision tree). Therefore, SHAP performs better overall in terms of local stability for both datasets compared to LIME.

#### 5.4.5. Efficiency

We next measure the runtime of SHAP and LIME for all the six models on both datasets. For each sample in the datasets, we record the time taken to compute the XAI explanation using each method (SHAP or LIME), which gives us the per-sample runtime. We then aggregate the runtime of different samples to show the global efficiency of each XAI method on many samples for our six models.

**Efficiency of VeReMi dataset (multiclass):** We start by the multiclass anomaly detection problem for the VeReMi dataset. We calculated the efficiency (amount of time required to generate explanation) for different sample numbers for SHAP and LIME for each of the six AI models. Here, for SHAP we considered sample sizes of 500, 1 k, 10 k, and 50 k. We also considered the same for LIME. Afterward, we compared the efficiency of SHAP and LIME for different samples across different models for comparing the efficiency of the two XAI methods. Table 10 summarizes the main results of the time efficiency of the different models for both SHAP and LIME. It shows that LIME performs better in terms of efficiency compared to SHAP for all models except SVM overall. However, with a lower number of samples SHAP performs better than LIME for some models. For DNN in SHAP, NA means not applicable, as for 50 k samples in DNN we do not have an estimated time for SHAP to produce any related time limit which is similar for SVM in LIME. Overall, LIME has better time efficiency in multiclass anomaly detection problem compared to SHAP for VeReMi.

**Efficiency of VeReMi dataset (binary class):** We next show the binary-class anomaly detection problem (i.e., all samples from different attack types are anomalous with the same label of 1, while normal samples have the label 0). For the efficiency of the VeReMi dataset for binary-class classification, we again conducted the efficiency experiment similar to the previous setup (as mentioned above for the multiclass problem). Table 11 shows the efficiency for both SHAP and LIME for all our AI models. Here, we also observe the same result that LIME performs better than SHAP overall except for 50 k samples in SVM in terms of runtime efficiency, with some minor exceptions in cases of lower sample numbers.

**Efficiency of Sensor dataset:** Similarly, we measure the efficiency of SHAP and LIME for all AI models for the Sensor dataset with the same setup as mentioned for the VeReMi dataset. The first difference is that here we only have two classes (normal and anomalous). The second difference for the Sensor dataset is that we have a testing with size of 3 k samples. Therefore, there are no rows for efficiency of 10 k or 50 k. Table 12 shows the main results for the experiment. We emphasize that in our evaluation results LIME performs better than SHAP for all AI models in terms of computational efficiency (runtime in minutes) for the Sensor dataset.

#### 5.4.6. Robustness

We next evaluate the robustness of our two XAI methods (SHAP and LIME). Robustness is an important evaluation criterion for explainable AI models. A model is said to be robust if adversarial perturbations cannot make changes to the outcome of XAI models [21]. Now, we perform the robustness test for our SHAP and LIME methods based on the method mentioned in [22] for the VeReMi and Sensor datasets. Here, we build a biased model (also known as racist model) and an adversarial model. The biased model reproduces an explanation for a signature attack, such an attack has a top feature that most characterizes it, and for the purposes of the experiment we called it the biased feature. In contrast, the adversarial model creates a fabricated explanation with a fake feature called ’unrelated’, such a feature is engineered to have an even higher correlation than the biased feature. Hence, the unrelated feature is bound to appear as the top feature in the fabricated explanation, misleading the analyst. For this experiment, “pos_x” is the biased feature for the VeReMi dataset, and “formality” is the biased feature for the sensor dataset. Then, we incorporate the (unrelated_column) when training the adversarial model to deceive SHAP and LIME. We emphasize that we leveraged the code mentioned in the prior work [22] for the robustness test to draw our different robustness results, detailed next.

**Robustness test:** We next show the evidence for a successful attack for both SHAP and LIME via the top features.

**Figure 8:**
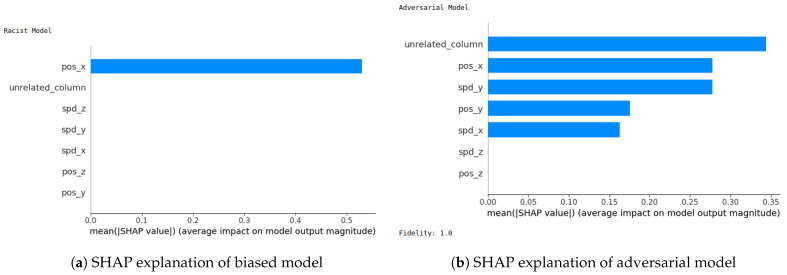
Robustness test for SHAP on VeReMi dataset. The adversarial model is successful in hiding the biased feature and letting SHAP explain the sample via the most significant feature “unrelated_column”, whereas in reality “pos_x” is the most significant feature.

**Figure 9:**
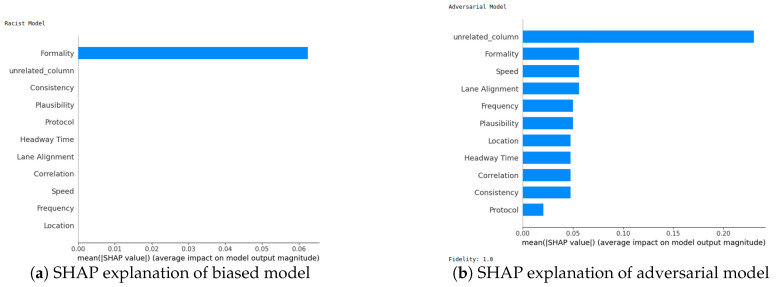
Robustness test for SHAP on Sensor dataset. The adversarial model deceives SHAP.

**(i)** 
**SHAP—VeReMi dataset:** For the robustness test of SHAP for the VeReMi dataset, the adversarial model is successful in hiding the biased feature and lets SHAP explain the sample via the most significant feature “unrelated_column”, whereas in reality “pos_x” is the most significant feature for this sample to reach the classification decision. Figure 8 shows this finding. The prediction fidelity is also “1”, which means that the prediction of the two models (biased and adversarial) is the same. Therefore, the adversarial model is successful in fooling the SHAP model.
**(ii)** 
**SHAP—Sensor dataset:** In the case of the Sensor dataset, the biased model is explaining that “Formality” is the major contributing role for this particular sample. However, Figure 9 shows that the adversarial model is successful in hiding the most important feature with the synthetic “unrelated_column” feature being the top feature under that adversarial model. Moreover, Fidelity is “1” which means the adversarial model is totally replicating the biased model on the same sample. Therefore, SHAP model was deceived here.

**Figure 10:**
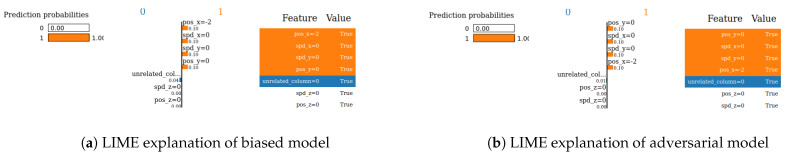
Robustness test for LIME on VeReMi dataset. After attack, the top feature is an existing feature (pos_y).

**LIME—VeReMi dataset:** For the robustness test of LIME for the VeReMi dataset, 0 means benign and 1 means anomalous class. Though the prediction fidelity is “1”, which means both models’ predictions match for this sample, it can be seen that the top feature is different (Figure 10). Here, the adversarial model also was able to replace the biased feature “pos_x” with “pos_y”. However, the adversarial model could not place the synthetic “unrelated_column” as the top feature for LIME. So, LIME was also fooled but not totally in this scenario (i.e., another feature from the dataset “pos_y” was the top feature not the unrelated one).

**Figure 11:**
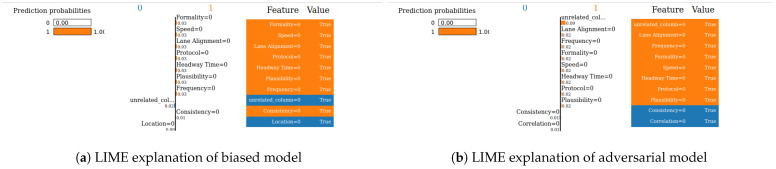
Robustness test for LIME on Sensor dataset. After attack, the top feature is the fabricated one (unrelated_column).

**LIME—Sensor dataset:** We finally show the robustness test for LIME on the Sensor dataset. Figure 11 shows the main findings of this test. It shows that even if both of the models’ predictions for this sample are correct and the prediction fidelity is “1”, the adversarial model is successful in masking the dataset’s biased feature—"formality"—with a fabricated “unrelated_column.” Thus, LIME was fooled completely in this experiment on the Sensor dataset by the adversarial model.

**Figure 12:**
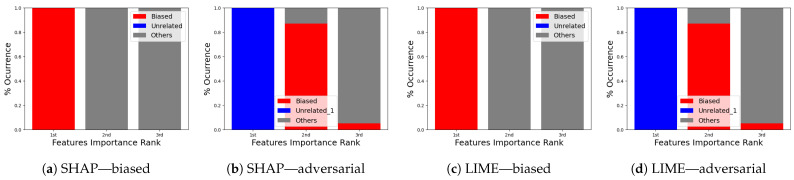
The percentage of data samples in the VeReMi dataset for which biased and unrelated features appear in the top 3 features (according to LIME and SHAP rankings of feature importance) for the biased classifier (in (**a**,**c**)) and adversarial classifier (in (**b**,**d**)) that uses one uncorrelated feature. We observe that the performance of both SHAP and LIME are almost same, as they bring out the biased feature in the top positions, which indicates that they used the biased feature to reach the decision.

**Figure 13:**
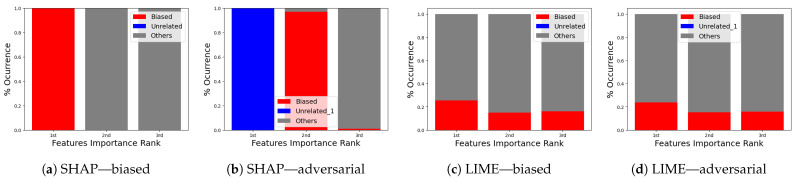
The percentage of data samples in the Sensor dataset for which biased and unrelated features appear in the top 3 features (according to LIME and SHAP rankings of feature importance) for the biased classifier (in (**a**,**c**)) and adversarial classifier (in (**b**,**d**)) that uses one uncorrelated feature. We observe that LIME exhibits higher resilience to adversarial attacks in the context of this Sensor dataset (shown by lower percentage of occurrence of unrelated feature in top three features for LIME).

**Occurrence percentage test:** The occurrence test consists of repeating the experiment shown in Figure 8 a hundred times to understand the occurrence behavior of which features appear as the top three for the biased and for the adversarial model. After completion, the results (Figure 12a–d and Figure 13a–d) are aggregated into three different stacks: “biased” if the biased feature appears (red), “unrelated” if the unrelated feature appears (blue), and “others” if any other feature appears (gray). Recall that the term “unrelated_column” refers to the column we introduced to the dataset to assess the resilience of the XAI methods against both biased and adversarial classifiers.

In the VeReMi dataset, we initially present the frequency with which each feature ranks among the top three in importance, as determined by LIME and SHAP. Notably, the biased model consistently places the biased feature at the top, as anticipated (Figure 12a,c). Conversely, the adversarial model (Figure 12b,d) reveals the unrelated feature as the most significant, with the biased feature frequently appearing second. In this scenario, the performance of both SHAP and LIME is nearly identical, consistently highlighting the biased feature in the second and third positions, regardless of the attacker’s efforts to suppress it. This suggests that the biased feature is instrumental in the decision-making process. As this is the feature the attacker aims to conceal, its consistent emergence underscores the resilience of the XAI method for this dataset. In summary, both LIME and SHAP exhibited comparable performance in this VeReMi dataset experiment, indicating their suitability for applications requiring robustness in feature occurrence. While the attack was partially successful, both SHAP and LIME consistently prioritized the biased features, providing analysts with an opportunity to identify the attack.

For the Sensor dataset, we obtain almost the same result for SHAP (Figure 13a,b). Despite the successful execution of the attack, SHAP managed to effectively identify and prioritize the biased feature, thereby providing analysts with an opportunity to detect and counter the attack. In contrast, it can be observed that LIME exhibits higher resilience to adversarial attacks in the context of this dataset. In Figure 13d, it is evident that the attackers were unable to conceal the presence of a biased feature with the “unrelated_column” feature within the adversarial model. Consequently, it becomes apparent that the attackers employed the biased feature to make a particular decision.

**Sensitivity of robustness:** To better grasp our robustness experiment, it is important to understand the concept of out-of-distribution (OOD) instances. In simple terms, OOD instances are created by making changes to the data and inserting noise data such that these samples do not come from the dataset we initially tested. Plus, Ref. [22] discusses a relationship between the ability to identify the OOD samples and the robustness. In other words, the higher the ability to differentiate the artificially OOD-generated samples from the original dataset samples, the more likely the attack will succeed. Following this statement, the work enlists OOD classifiers that are trained to identify the OOD samples, with the chosen metric being the F1 score.

**Figure 14:**
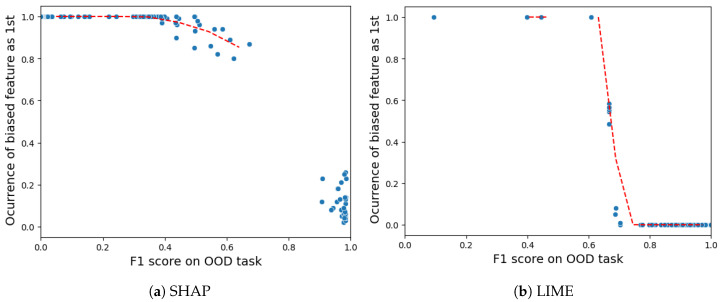
Robustness sensitivity for SHAP and LIME for the VeReMi dataset. We observe that LIME is more robust than SHAP.

For the VeReMi dataset, Figure 14a shows that SHAP is resistant to attacks up to an F1 score of 0.4, then it starts to present a gradual fall until an x-axis value of 0.7, indicating risk of vulnerability. Between the F1 values of 0.7 and 0.9 there is a gap in the data available, indicating there are no results for these values, and after the F1 value of 0.9 SHAP is highly vulnerable to attacks. However, for LIME Figure 14b shows that it is more robust till the threshold of the F1 score surpasses 0.6. After that, LIME experiences a sharp and steep fall, making it more vulnerable to attacks. Overall, LIME performs better in this sensitivity experiment as it is more robust up to a threshold of 0.6 and SHAP starts to show signs of vulnerability at a threshold of 0.4. So in essence, the attack relies on accurately identifying perturbed data points to “fool” the XAI explanations on those points while retaining bias on real data. If perturbation detection is poor, there is a high chance that the attack will fail. On the other hand, reasonably accurate detection enables effective hiding of bias from explanations, with LIME demanding an OOD F1 score higher than SHAP.

**Figure 15:**
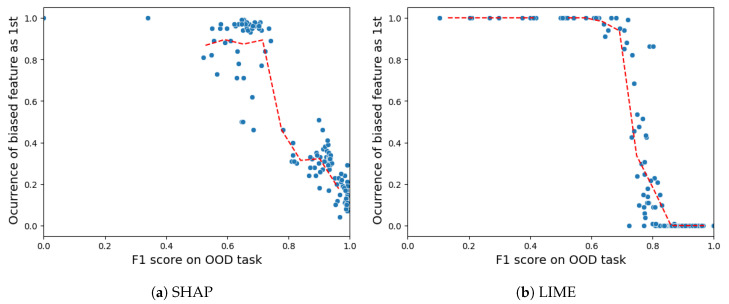
Robustness sensitivity for SHAP and LIME for Sensor dataset. We observe that LIME is more robust than SHAP.

For the Sensor dataset, Figure 15 shows that both LIME and SHAP exhibit a threshold value of approximately 0.7 for the F1 score on the out-of-distribution (OOD) task. Once the threshold of 0.7 is surpassed, both the SHAP and LIME methods exhibit significant vulnerability to adversarial attacks. However, LIME demonstrates superiority due to its ability to endure almost all attacks below an OOD F1 score of 0.8, while SHAP still suffers attacks with occurrence values in the range of 0.1–0.4 above an OOD F1 score of 0.8.

#### 5.4.7. Completeness

Recall that an XAI method is considered complete when it is capable of delivering accurate explanations for all potential input samples, including corner samples. This concept served as the driving force behind our experiments to achieve completeness. For the completeness experiment, we adhered to the subsequent procedure for calculating the completeness score.

- Scale the data between 0 to 1 using MinMax scaler [55].
- Generate an explanation for each autonomous driving sample and check its explanation.
- Perturb the top five features (change their values from 0 to 1).
- Check if the predicted class changes after perturbations.
- If class does not change even after substantial perturbations, we conclude that the XAI explanation was not complete, i.e., the original explanation is not relevant or degenerated.

To summarize the goal of our experiment, a complete XAI method should satisfy the following conditions. First, the predicted class should change when the top influential features are perturbed. If the class does not change, those features may not be that influential, which questions the explanation’s validity. Second, all types of samples, including outliers/corner cases, should have valid explanations where class changes according to feature perturbations. Lack of valid explanations for some samples indicates incompleteness of the XAI method. We next summarize our results.

**Figure 16:**
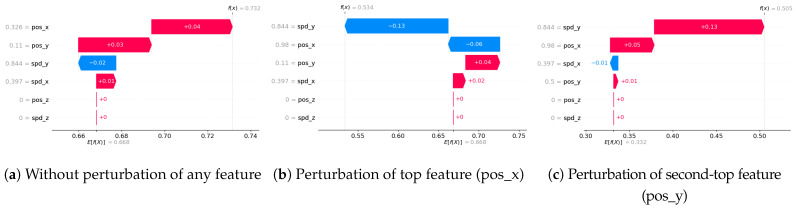
Local completeness of SHAP for the VeReMi dataset using a benign sample using RF model. Red and blue colors show positive or negative contribution of the features, respectively.

**Local completeness:** For local analysis, the methodology involves taking an example instance (e.g., a sample classified as an anomalous AV) and incrementally perturbing the top features from its SHAP/LIME explanation. After each perturbation, the sample’s predicted class is checked, with the process continuing until the class prediction flips to normal AV. If the class fails to change despite significant feature perturbations, it implies that the original explanation was incomplete and not relevant in capturing the model’s reasoning. For the VeReMi dataset, Figure 16 shows the local completeness test for a single benign sample for SHAP. Figure 16a depicts the features according to SHAP importance from top to bottom. Then, we manually perturb the top feature “pos_x” and change its SHAP value to 1. Even though the perturbation is the maximum (the value of the feature’s perturbation is 1), the sample does not change its class (as shown in Figure 16b). Then, we perturb the second most influential feature, “pos_y”, to 0.5. After that, this sample changes its class, showing that it is complete.

**Figure 17:**
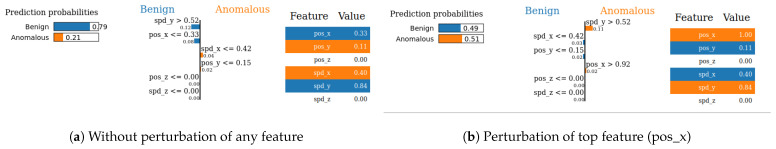
Local completeness of LIME for the VeReMi dataset. LIME flips the predicted class after only perturbing the top feature.

Figure 17 delineates the local completeness of LIME for a single benign sample of the VeReMi dataset. Figure 17a shows the list of the top features and their contribution without any external perturbation. Now, we perturb the most influential feature for this sample, which is “pos_x”, from 0.33 to 1. As we do that, Figure 17b shows that the sample has become anomalous as the prediction probability for this sample has increased and surpassed 50%, indicating this sample is complete. Overall, for this local sample LIME performs better compared to SHAP since LIME requires only one feature perturbation to change its class whereas SHAP requires two perturbations of two features to change the predicted class.

**Figure 18:**
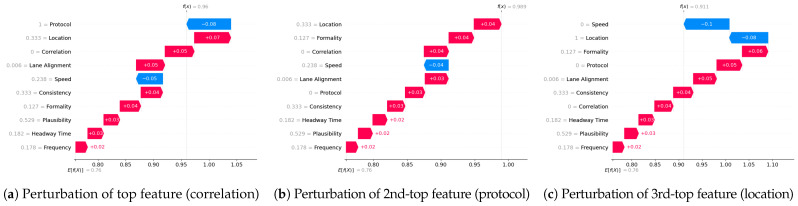

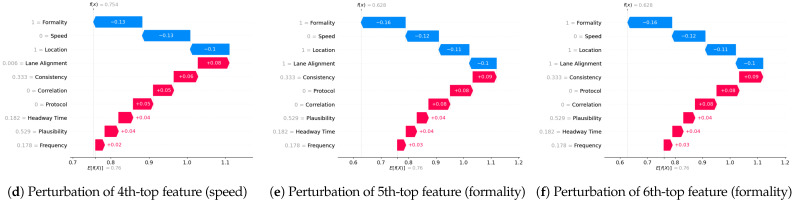
Local completeness of SHAP for Sensor dataset. Altering the values of the top six features induces a class change. Red and blue colors show positive or negative contribution of the features.

**Figure 19:**
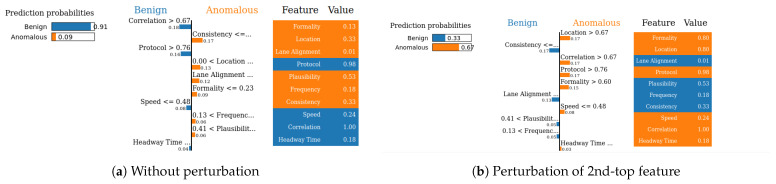
Local completeness of LIME for Sensor dataset. Altering the values of the top two features induces a class change.

For the local completeness experiment conducted on the Sensor dataset, we observe that altering the values of the top six features was necessary to induce a change in the class label for SHAP. Figure 18 shows the perturbations of the features it required to change its class. In the case of LIME, Figure 19 shows that perturbing only the top two features led to a change in the predicted class. Therefore, LIME performs better than SHAP in terms of local completeness for the Sensor dataset as well.

**Global completeness:** Global completeness extends the aforementioned completeness analysis across thousands of samples per class. The top five explanatory features are perturbed for the entire batch of 1000 samples, and the proportion of samples that alter classification is recorded. Lower percentages of class changes indicates a greater prevalence of incompleteness, with the XAI methods providing invalid rationales for those samples’ predictions.

**Figure 20:**
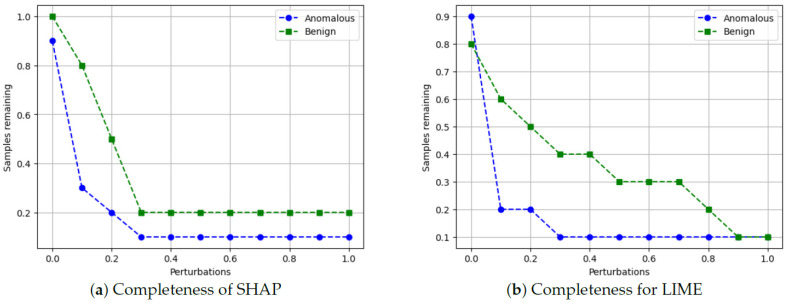
Completeness for both XAI methods (SHAP and LIME) for the VeReMi dataset. We observe that SHAP has better explanations but LIME was able to explain 10% more samples than SHAP for the benign class.

**Figure 21:**
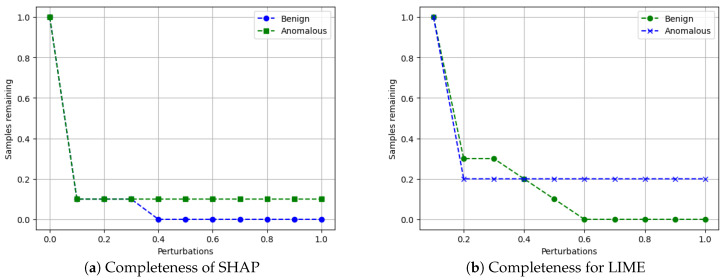
Completeness for both XAI methods (SHAP and LIME) for Sensor dataset. We observe that SHAP outperformed LIME in terms of completeness for this dataset due to its earlier convergence to the x-axis, smaller area under the curve, and fewer remaining samples.

**Main intuitions:** Figure 20 and Figure 21 convey the same information through a different perspective to Table 13 and Table 14. In depth, the y-axis of Figure 20 and Figure 21 “samples remaining” refers to remaining samples that were not able to achieve a valid explanation after the perturbation applied in the x-axis “perturbations”. The rationale indicates that as the number of perturbations increases, a drop in the remaining samples is expected because perturbations in the most important feature values cause a change in class in the expected scenario. Therefore, as more samples are checked to have valid explanations, the number of remaining samples without a valid explanation tends to decrease. Such graphs aid the intuition of the quality of explanations generated by the XAI regarding its resistance to change. The best-case scenario from the explainability perspective is to have higher decreases with fewer perturbations. Also, if the trend does not reach zero on the y-axis, it means that there are remaining samples which are without a valid explanation. As a general rule, to see which one is better we should check which one has less area under the graph (or has the sharpest falls).

For the VeReMi dataset, the results in Table 13 and Figure 20 reveal that SHAP and LIME are incomplete since they are unable to give complete explanations for all the classes. This suggests that even after making significant changes to the five top attributes, SHAP and LIME were unable to modify the predicted class. On the other hand, if the original top five features were actually relevant, they should have had a significant impact on the model’s forecast, but this did not occur. Although both SHAP and LIME are incomplete, LIME outperforms SHAP as it generates 90% benign samples and 90% of anomalous samples completely, whereas SHAP generates only 80% of benign samples completely.

For the Sensor dataset, Table 14 and Figure 21 provide an overview of the global completeness percentages for the Sensor dataset. These percentages represent the number of complete samples out of a total of 1000, categorized into benign (0) and anomalous (1) classes. Although none of the XAI methods are fully comprehensive, it is seen that SHAP outperforms LIME in the context of the anomalous class, exhibiting a 10% advantage over LIME. The experiments also show that SHAP performs better regarding the quality of explanations in Figure 20 and Figure 21 due to the steep decrease in the curves, but LIME outperforms SHAP in the results shown in Table 13 due to achieving a higher percentage of valid explanations for the benign class.

Overall, our aforementioned experiments in evaluating completeness on both granular individual instances and broadly across group of samples reveal the different scopes of completeness for the SHAP and LIME XAI methods. Ultimately, comprehensive and accurate interpretability requires the XAI explanations to completely capture model logic across the entirety of the anomaly detection problem space.

**Summary of results:** For evaluating the performance of two XAI methods, namely, SHAP and LIME, we present a summary of the results obtained through our proposed framework. This framework encompasses six distinct evaluation metrics, each carrying equal weight in assessing the capabilities of the XAI models. Consequently, the maximum attainable score for any given XAI model is six, representing a successful fulfillment of all evaluation criteria. From an ablation perspective, the scoring system operates on a binary scale, where a score of one is assigned to a particular XAI model if it passes a specific evaluation metric, and a score of zero is assigned if it fails to meet the criteria. This approach allows for a quantitative assessment of the relative strengths and weaknesses of each XAI model across the different evaluation dimensions. The ablation experiment (Table 15) reveals the distinct contributions of SHAP and LIME to the model’s performance. SHAP, with a total score of 3, significantly enhances descriptive accuracy, sparsity, and stability, indicating its vital role in providing concise, consistent explanations aligned with decision-making processes. Conversely, LIME, with a total score of 2, excels in efficiency and robustness, offering faster explanations and reliability under noise or adversarial conditions. Removing SHAP would diminish the model’s accuracy, focus, and consistency, while removing LIME would reduce its computational efficiency and robustness. This analysis underscores the unique strengths of each model, guiding informed decisions on their integration and optimization based on application-specific needs.

### 5.5. Ablation Experiments

We now perform ablation tests on the AI models and feature normalization approaches to clarify their individual contributions to overall performance. By removing the top-performing models, we can assess the specific influence of these leading designs on benchmark tasks. Similarly, when feature normalization is removed, it becomes clear how necessary preprocessing steps are for achieving optimal results.

**Ablation of top AI models:** Now, we consider the part where we remove the top performing AI models from each of our datasets (VeReMi and Sensor) for SHAP and LIME and analyze the descriptive accuracy. This analysis aims to comprehend the vital role played by the best-performing models in the overall accuracy of the respective datasets. The methodology involves calculating the average accuracy across all six AI models as a baseline. Subsequently, the top-performing model is removed, and the average accuracy is recalculated using the remaining five models. By comparing the two averages, the decline in accuracy can be quantified, providing insights into the contribution of the top-performing model to the overall performance. For the VeReMi dataset, the top-performing model identified is RF. Upon removing RF and averaging the accuracy of the remaining models, a decrease of 1% is observed (Table 16). Similarly, for the Sensor dataset, the top-performing model is ADA. When ADA is removed and the overall accuracy is recomputed, a more substantial drop of 3% is observed (Table 17). The results indicate that the top-performing models play a significant role in maintaining the overall accuracy of their respective datasets. However, the magnitude of their contribution varies, with the top model in the Sensor dataset having a more substantial impact compared to the VeReMi dataset.

**Ablation of feature normalization:** Now we provide the ablation effect of feature normalization for both of our datasets. In other words, what is the performance of each of our six AI models with feature normalization and without feature normalization? An ablation study on feature normalization reveals its varying impact on different machine learning models and datasets. In the VeReMi dataset (Table 18), feature normalization substantially benefits models like the DNN, which shows improvements in three out of four evaluation metrics—accuracy increases from 0.33 to 0.65, precision from 0.33 to 0.67, and F1 score from 0.50 to 0.79. A slight increase in the accuracy of KNN is also noticed, indicating that these models may be more sensitive to feature scaling.

For the Sensor dataset (Table 19), normalization consistently enhances performance for most models. The DT and RF models see marked improvements in accuracy. KNN’s accuracy rises dramatically from 0.78 without normalization to 0.82 with normalization. ADA maintains high performance across both scenarios, indicating its robustness to feature scaling.

These results suggest that while normalization is generally beneficial, especially for neural networks and tree-based models, its necessity and impact can vary, highlighting the importance of dataset-specific and model-specific considerations in preprocessing steps.

## 6. Limitations and Discussion

**(1)**  **Multiclass XAI evaluation:** In our paper, we primarily give complete treatment of evaluating XAI methods and their explanations for two datasets, with the main focus on the binary-class anomaly detection classification problem (although having some results for multiclass anomaly classification problems). However, we leave complete treatment of evaluating XAI explanations for multiclass classification for future works. For instance, identifying the different types of potential anomalies in VANETs may require further research. In this context, our proposed XAI evaluation methods can be also leveraged to test the actual contribution of different features for the multiclass anomaly detection problem. However, we leave a more thorough analysis of the multiclass XAI interpretation for autonomous driving for future research works.
**(2)**  **Exploring our XAI evaluation framework on other benchmark datasets:** We tested our XAI evaluation framework on two different datasets: the VeReMi [23] and Sensor [24,52] datasets. The VeReMi dataset focuses on detecting anomalies of an AV based on the position and speed of the AV while the Sensor dataset considers other communication-based data that the sensors mounted on AVs collect to detect anomalies. Nevertheless, there are other autonomous driving datasets (e.g., nuScenes [63], A2D2 [64], and Pass [65]) with other features that are not considered in our studied datasets. However, we emphasize that our proposed XAI evaluation framework can be leveraged to identify the effectiveness of XAI methods on these datasets and the contribution of different features for different types of datasets to enhance security of AVs. In addition, some of these datasets consider online anomaly detection. We highlight that our framework can be adapted to online anomaly detection, where our framework will accept instantaneous readings as inputs and provide classification and accompanying explanations and XAI evaluation metrics.
**(3)**  **Reliability of current black-box XAI methods:** Although XAI methods (particularly SHAP and LIME) can be used by auditors and safety drivers when gathering information and understanding logs from autonomous vehicles and accompanying networks, our work shows that the performance of SHAP and LIME would need to be improved to be used in real-world anomaly detection for autonomous driving systems. In particular, our work shows that it is desirable to enhance SHAP and LIME to be more robust against adversarial attacks. Furthermore, our analysis shows the need to validate the completeness of the explanations from SHAP and LIME before deploying them in reality in a safety-critical application like autonomous driving. To achieve such a goal, we shared our source codes to build on our framework with more models and datasets.
**(4)**  **Leveraging GNN insights for enhancing XAI in autonomous vehicle networks:** We used our XAI basically for the detection and evaluated the XAI methods as to whether they can be trusted, without considering the network-level aspects and dynamic topologies of vehicular communication networks. Leveraging insights of graph neural networks (GNNs) in capturing network topologies and optimizing communication networks, the XAI framework for anomaly detection in AVs could be enhanced by incorporating network-level information, exploring GNN-based XAI techniques to better capture relational aspects, adapting to dynamic network topologies, and enabling multi-objective optimization beyond anomaly detection, while evaluating the trustworthiness of the XAI methods [66].

## 7. Conclusions

The usage of explainable AI (XAI) methods can help in improving the interpretability of AI models for the anomaly detection problem for autonomous driving systems (such as VANETs). The goal of such a usage is to make the classification decisions made by the black-box AI models understandable to human operators in the autonomous driving domain, and to take necessary precautions by identifying the significant features contributing to possible anomalies. In this paper, we presented an end-to-end framework for evaluating XAI techniques applied to the task of anomaly detection in autonomous vehicles. Our framework enables analyzing both global and local XAI methods for understanding decisions made by XAI methods which explain AI models that classify AV behavior. We analyzed six different evaluation metrics (descriptive accuracy, sparsity, stability, efficiency, robustness, and completeness) for two popular black-box XAI techniques, SHAP and LIME. We evaluated our XAI evaluation framework using two popular autonomous driving datasets (VeReMi and Sensor) while considering six different AI models. In our evaluation, we first used XAI techniques to extract the main features for anomaly classification. We then performed extensive experiments to evaluate SHAP and LIME according to the six different evaluation metrics on the two datasets. This work represents a critical step towards applying black-box XAI methods for real-world anomaly detection in autonomous driving systems via understanding the strengths and limitations of current black-box XAI methods when applied to that critical domain. The insights from our work will be useful in enhancing security of autonomous driving systems. Future avenues of related research would be evaluating our framework on diverse autonomous driving datasets, proposing XAI evaluation frameworks for white-box XAI methods, and further exploring XAI application on multiclass anomaly detection for autonomous driving.

## Figures and Tables

**Table 1:** Main sensors used for anomaly detection task of each autonomous vehicle.

Sensor Name	Normal Data Range	Description
Formality	1–10 bit	Checks every message for if it is maintaining correct formality
Location	0/1	Checks if the message reached the destined location
Frequency	1–10 Hz	Checks the interval time of messages
Speed	50–90 mph	Checking if the AV is within the speed limit (highway)
Correlation	0/1	Checks if several messages adhere to defined specification
Lane Alignment	1–3	Checks if the AV is in the lane of the platoon
Headway Time	0.3–0.95 s	Checks if the AV maintains the headway time range
Protocol	1–10,000	Checks for the correct order of communication messages
Plausibility	50–200%	Checks if the data are plausible (relative size difference between two consecutive payloads)
Consistency	0/1	Checks if all the parts of the AV are delivering consistent information about an incident

**Table 2:** Description of the main features of VeReMi dataset.

Column	Description
pos_x	The x-coordinate of the vehicle position
pos_y	The y-coordinate of the vehicle position
pos_z	The z-coordinate of the vehicle position
spd_x	The speed of the vehicle in x-direction
spd_y	The speed of the vehicle in y-direction
spd_z	The speed of the vehicle in z-direction

**Table 3:** Statistics of both VeReMi and Sensor datasets.

Parameter	VeReMi Dataset	Sensor Dataset
Labels	5	2
Number of Features	6	10
Dataset Size	993,834	10,000
Training Sample	695,684	7000
Testing Sample	298,150	3000
Normal Samples No.	664,131	5000
Anomalous Samples No.	329,703	5000

**Table 6:** Global stability of SHAP and LIME across the six AI models for the VeReMi dataset. SHAP has better stability compared to LIME.

XAI Methods	DT	RF	DNN	KNN	SVM	ADA
SHAP	0.67	0.33	0.67	0.33	1.00	1.00
LIME	0.67	0.67	0.33	0.33	0.67	0.67

**Table 7:** Global stability of SHAP and LIME across the six AI models for the Sensor dataset. SHAP has better stability.

XAI Methods	DT	RF	DNN	KNN	SVM	ADA
SHAP	0.33	0.33	1.00	1.00	0.67	1.00
LIME	0.33	0.67	0.67	1.00	0.33	0.33

**Table 8:** Local stability of SHAP and LIME across the six AI models for the VeReMi dataset. SHAP has better local stability.

XAI Methods	DT	RF	DNN	KNN	SVM	ADA
SHAP	0.67	1.00	1.00	0.67	1.00	0.67
LIME	0.67	0.67	1.00	0.67	0.67	0.67

**Table 9:** Local stability of SHAP and LIME across the six AI models for the Sensor dataset. SHAP has better local stability.

XAI Methods	DT	RF	DNN	KNN	SVM	ADA
SHAP	0.40	0.40	1.00	1.00	1.00	1.00
LIME	0.60	0.40	0.80	0.20	0.60	0.80

**Table 10:** Efficiency (runtime in minutes) of XAI methods (SHAP and LIME) for different AI models on VeReMi dataset (multiclass problem). We observe that LIME has better efficiency compared to SHAP for all models except SVM.

XAI Model	# Samples	DT	RF	DNN	KNN	SVM	ADA
SHAP	500	0.87	1.87	0.29	2.71	60.18	1.30
	1 k	3.26	6.80	0.47	10.53	264.45	4.85
	10 k	401.91	662.81	22.53	1259.01	28,087	437.75
	50 k	7159.65	18,735.48	NA	29,902.33	26,201.26	14,101.28
LIME	500	0.11	2.49	2.9	3.45	72.96	0.45
	1 k	0.22	2.96	7.71	2.91	84.62	1.04
	10 k	4.18	33.03	57.88	55.11	NA	15.39
	50 k	20.56	136	289.80	197.4	NA	70.9

**Table 11:** Efficiency (runtime in minutes) of XAI methods (SHAP and LIME) for different AI models on VeReMi dataset (binary classification problem). Again, we observe that LIME has better efficiency than SHAP for the majority of AI models and under different numbers of samples.

XAI Model	# Samples	DT	RF	DNN	KNN	SVM	ADA
SHAP	500	0.72	1.84	0.13	2.47	19.09	1.13
	1 k	2.97	6.29	0.15	9.06	93.66	4.15
	10 k	219.66	535.31	6.98	929.98	6711.26	477.23
	50 k	8054.65	13,256.81	NA	22,128.2	271,275	8828.6
LIME	500	0.12	2.48	4.65	4.94	25.53	1.04
	1 k	0.43	3.11	5.66	5.16	24.71	0.65
	10 k	27.24	43.1	95.90	89	379.38	18.88
	50 k	27.23	131.88	501.67	287.75	NA	56.41

**Table 12:** Efficiency (runtime in minutes) of XAI methods for different AI models on Sensor dataset. LIME has better efficiency for the Sensor dataset compared to SHAP.

XAI Models	No. of Samples	DT	RF	DNN	SVM	KNN	ADA
SHAP	500	0.15	0.67	169.3	588	369	0.6
	1 k	34	143	692	1915	1209	104
	3 k	409	1327	6057	22,677	12,721	1202
LIME	500	0.27	0.84	3.31	5.36	3.42	0.65
	1 k	0.77	2.17	7.32	10.75	7.19	1.71
	3 k	2.4	6.51	20.13	32.77	21.97	5.07

**Table 13:** The percentage of samples that are complete for each class using VeReMi dataset. We observe that both SHAP and LIME are incomplete since they are unable to give complete explanations for all the classes.

XAI Methods	Benign (0)	Anomalous (1)
SHAP	80%	90%
LIME	90%	90%

**Table 14:** The percentage of samples that are complete for each class using Sensor dataset. We observe that SHAP has better performance compared to LIME in providing complete explanations for anomalous samples.

XAI Methods	Benign (0)	Anomalous (1)
SHAP	100%	90%
LIME	100%	80%

**Table 15:** A summary of performance comparison between SHAP and LIME for all six performance metrics. Overall, SHAP provides better performance compared to LIME for our six metrics.

XAI Models	Descriptive Accuracy	Sparsity	Stability	Efficiency	Robustness	Completeness	Total Score
SHAP	1	1	1	0	0	0	3
LIME	0	0	0	1	1	0	2

**Table 16:** Drop in average accuracy when removing the top-performing AI model (RF). We observe that after removing RF, the average accuracy drops by 1% for the VeReMi dataset.

XAI Method		DT	RF	DNN	KNN	SVM	ADA	Average
SHAP	Acc	0.75	**0.80**	0.61	0.78	0.67	0.69	0.71
	Acc	0.75	✗	0.61	0.78	0.67	0.69	0.70
LIME	Acc	0.75	**0.80**	0.61	0.78	0.67	0.69	0.71
	Acc	0.75	✗	0.61	0.78	0.67	0.69	0.70

**Table 17:** Drop in average accuracy when removing the top-performing AI model (ADA). We observe that after removing ADA, the average accuracy drops by 3% for the Sensor dataset.

XAI Method		DT	RF	DNN	KNN	SVM	ADA	Average
SHAP	Acc	0.85	0.90	0.55	0.84	0.88	0.99	0.83
	Acc	0.85	0.90	0.55	0.84	0.88	✗	0.80
LIME	Acc	0.85	0.90	0.55	0.84	0.88	0.99	0.83
	Acc	0.85	0.90	0.55	0.84	0.88	✗	0.80

**Table 18:** Ablation effect of feature normalization on VeReMi dataset. We observe the highest improvement for the DNN model when the features are normalized.

Evaluation Metric	Normalized						Not Normalized					
	**DT**	**RF**	**DNN**	**KNN**	**SVM**	**ADA**	**DT**	**RF**	**DNN**	**KNN**	**SVM**	**ADA**
Acc	0.78	0.80	0.65	0.78	0.67	0.73	0.79	0.80	0.33	0.79	0.67	0.73
Prec	0.82	0.82	0.67	0.82	0.67	0.75	0.82	0.82	0.33	0.83	0.69	0.75
Rec	0.87	0.91	0.96	0.85	1.00	0.91	0.87	0.91	1.00	0.85	0.79	0.91
F1	0.84	0.86	0.79	0.84	0.80	0.82	0.84	0.86	0.50	0.84	0.79	0.82

**Table 19:** Ablation effect of feature normalization on Sensor dataset. We observe the highest improvement for the KNN model when the features are normalized.

Evaluation Metric	Normalized						Not Normalized					
	**DT**	**RF**	**DNN**	**KNN**	**SVM**	**ADA**	**DT**	**RF**	**DNN**	**KNN**	**SVM**	**ADA**
Acc	0.84	0.90	0.49	0.82	0.83	1.00	0.85	0.91	0.67	0.78	0.83	1.00
Prec	0.89	0.90	0.76	0.82	0.85	1.00	0.89	0.91	0.79	0.79	0.85	1.00
Rec	0.91	0.97	0.49	0.99	0.95	1.00	0.92	0.98	0.77	0.96	0.94	1.00
F1	0.90	0.94	0.60	0.90	0.89	1.00	0.90	0.95	0.78	0.87	0.89	1.00

## Data Availability

We adhere to the data availability policy outlined by MDPI journals. The data supporting the findings of this study are available in the public repository at the following URL: https://github.com/Nazat28/EXAI_ADS (accessed on 25 May 2024). For inquiries regarding specific datasets or further clarification, please contact the corresponding author.

## Appendix A. Hyperparameters of AI Models

We now present the hyperparameter configurations for our different AI models.

(1) **Decision tree (DT):** Decision tree classifier was our first AI model that we experimented with on the datasets. The best hyperparameter choice for this model was when the criterion was set to “gini” for measuring impurity and the max depth of the tree was 50, which means the number of nodes from root node to the last leaf node was 50. The minimum number of samples required to be present in a leaf node was 4 (min_samples_leaf=4) and the minimum number of samples required to split a node into two child nodes was 2 (min_samples_split=2). To have reproducibility and testing on the same data we set random_state to 100 and the rest of the hyperparameters were set as default.
(2) **Random forest (RF):** We next implemented RF classifier, where max_depth was set to 50. The number of estimators (number of decision trees) was set to 100, min_samples_leaf was set to 1, min_samples_split was the same as that of DT.
(3) **Deep neural network (DNN):** We next show the best values for the DNN classifier. We set the dropout value to 0.1, and added one hidden layer with a size of 16 neurons with rectified linear unit (ReLU) as the activation function. Next, we set the optimization algorithm as ‘Adam’ and the loss function was set to “binary_crossentropy”. The epochs were set to 5, with a batch size of 100 to train the DNN model. We set the rest of the hyperparameters as given by the default configuration.
(4) **K-nearest neighbours (KNN)**: The parameters we set for KNN were as follows: the number of neighbors was set to 5 (n_neighbors=5), the leaf size was set to 30 to speed up the algorithm, the distance metric was set to “minkowski” to compute distance between neighbors, and the search algorithm for this model was “auto”.
(5) **Support vector machine (SVM)**: For the SVM AI classifier, we set the regularization parameter to 1 (C = 1). The kernel function was set to radial basis function (RBF). The kernel coefficient to control the decision boundary was set to “auto”. All other hyperparameters were used as they were provided by default.
(6) **Adaptive boosting (ADA)**: Finally, the hyperparameters we used for AdaBoost were as follows: “base_estimator” was set to ‘DecisionTreeClassifier’ with a maximum depth of 50. The number of estimators was 200 and the “learning_rate” was 1. The boosting algorithm was set to “SAMME.R” to converge faster and to achieve a lower test error.

## Appendix B. Tuning of Hyperparameters for Our Datasets

**VeReMi dataset:** In evaluating six of our classifiers on the VeReMi dataset, a comprehensive analysis reveals distinctive impacts of hyperparameter tuning on their performance metrics. Table A1 shows the DT model exhibited enhanced performance metrics, including an accuracy of 0.79, precision of 0.82, recall of 0.87, and F1-score of 0.85, achieved through adjustments in max_depth = 100 and min_samples_leaf = 4. Similarly, the RF model demonstrated notable improvements with an accuracy of 0.80, precision of 0.83, recall of 0.88, and F1-score of 0.86, primarily attributed to an increased n_estimator = 100. In contrast, the deep neural network (DNN) and support vector machine (SVM) models, despite efforts to optimize hyperparameters, yielded comparatively lower metrics, with the best DNN model achieving an accuracy of 0.67, precision of 0.67, recall of 1.00, and F1-score of 0.80, and the SVM model exhibiting similar recall but lower precision and accuracy (accuracy: 0.67, precision: 0.67, recall: 1.00, F1-score: 0.80). Conversely, the AdaBoost (ADA) model with adjusted max_depth = 100 showed competitive performance metrics, including an accuracy of 0.79, precision of 0.81, recall of 0.82, and F1-score of 0.85, thereby outperforming the RF model in precision and F1-score. Furthermore, the K-nearest neighbors (KNN) model demonstrated promising results, achieving an accuracy of 0.78, precision of 0.84, recall of 0.85, and F1-score of 0.84, albeit marginally lower than the optimal RF model. Notably, the AdaBoost classifier with base_estimator of DecisionStump served as a baseline, highlighting potential class imbalance or triviality within the dataset, exhibiting an accuracy of 0.87, precision of 0.89, recall of 0.89, and F1-score of 0.88. In conclusion, the AdaBoost model, fine-tuned with base_estimator = DecisionStump, emerged as the top-performing classifier for the VeReMi dataset, closely followed by the RF model, while the DT and KNN models also showed competitive performance post-hyperparameter optimization. Nonetheless, the DNN and SVM models displayed inferior performance compared to the tree-based models.

**Sensor dataset:** We now provide hyperparameter tuning for our six AI models (DT, RF, DNN, KNN, SVM, and ADA) on the Sensor dataset, which allows us to compare the performance of each model under different hyperparameters. From Table A2 we see that for DT, increasing the maximum depth (max_depth) and minimum number of samples required to be a leaf node (min_samples_leaf) generally improved the performance metrics, particularly precision and F1-score, while maintaining a good balance between recall and accuracy. The best DT model achieved an accuracy of 0.86, precision of 0.89, recall of 0.93, and F1-score of 0.91 with max_depth = 100 and min_samples_leaf = 4. Secondly, for RF, increasing the number of estimators (n_estimators) consistently improved the performance metrics, with the best results obtained with n_estimators = 100. And the best RF model achieved an accuracy of 0.91, precision of 0.91, recall of 0.98, and F1-score of 0.94, outperforming the tuned DT model in all metrics except recall. For DNN, increasing hidden_layer from 1 to 3 resulted in a decline in all the evaluation metrics. For KNN, varying the number of neighbors (n_neighbors) and leaf size (leaf_size) had a moderate impact on the performance metrics of KNN. Tweaking n_neighbors from 3 to 10 resulted in a moderate increase in all the evaluation metrics. Changing the SVM ’kernel’ from ’linear’ to ’rbf’ and ’regularization’ from 10 to 1 parameter significantly affected performance; the optimal model achieved an accuracy of 0.88, precision of 0.90, recall of 0.95, and F1-score of 0.92. Lastly, when the base_estimator was changed from DecisionTreeClassifier to DecisionStump, it performed the best among all the models with a perfect score of 1.00 for all the evaluation metrics.

**Table A1:** Hyperparameters tuning effect on VeReMi dataset. We observe that AdaBoost performs the best in all the evaluation metrics when DecisionStump is used as the base_estimator.

	DT						RF						DNN					
	**max_depth**				**min_samples_leaf**		**max_depth**				**n_estimator**		**hidden_layers**				**Epochs**	
	4	50		100	4	20	4	50		100	100	200	1		3		5	10
Acc	0.67	0.79		0.79	0.79	0.75	0.67	0.80		0.80	0.80	0.80	0.40		0.67		0.57	0.67
Prec	0.67	0.82		0.82	0.82	0.78	0.67	0.83		0.82	0.83	0.83	0.69		0.67		0.67	0.67
Rec	**1**	0.87		0.87	0.87	0.86	**1**	0.88		0.87	0.88	0.88	0.19		**1**		0.70	**1**
F1	0.80	0.84		0.85	0.84	0.82	0.80	0.86		0.83	0.86	0.85	0.39		0.80		0.69	0.80
	**KNN**						**SVM**						**ADA**					
	**n_neighbors**				**leaf_size**		**C**				**kernel**		**max_depth**				**Classifier**	
	3		10		5	50	1		10		rbf	linear	4	50		100	DT	DS
Acc	0.78		0.75		0.78	0.78	0.67		0.67		0.67	0.67	0.70	0.79		0.79	0.79	**0.87**
Prec	0.84		0.78		0.82	0.83	0.69		0.67		0.67	0.67	0.71	0.84		0.81	0.84	**0.89**
Rec	0.84		0.88		0.85	0.85	0.92		**1**		0.92	**1**	0.93	0.85		0.82	0.85	0.89
F1	0.84		0.83		0.84	0.84	0.78		0.80		0.80	0.80	0.81	0.85		0.85	0.85	**0.88**

**Table A2:** Hyperparameters tuning effect on Sensor dataset. We observe that AdaBoost performs the best in all the evaluation metrics when DecisionStump is used as the base_estimator.

	DT						RF						DNN					
	**max_depth**				**min_samples_leaf**		**max_depth**				**n_estimator**		**hidden_layers**				**Epochs**	
	4	50		100	4	20	4	50		100	100	200	1		3		5	10
Acc	0.81	0.85		0.86	0.85	0.84	0.82	0.91		0.91	0.91	0.90	0.55		0.49		0.55	0.49
Prec	0.83	0.89		0.89	0.89	0.88	0.81	0.91		0.91	0.91	0.91	0.80		0.76		0.80	0.76
Rec	0.94	0.92		0.93	0.92	0.93	0.99	0.98		0.98	0.98	0.97	0.55		0.49		0.55	0.49
F1	0.89	0.90		0.91	0.90	0.90	0.89	0.94		0.94	0.94	0.94	0.65		0.60		0.65	0.60
	**KNN**						**SVM**						**ADA**					
	**n_neighbors**				**leaf_size**		**C**				**kernel**		**max_depth**				**Classifier**	
	3		10		5	50	1		10		rbf	linear	4	50		100	DT	DS
Acc	0.83		0.84		0.83	0.82	0.88		0.81		0.88	0.81	0.99	0.85		0.85	0.99	**1.00**
Prec	0.84		0.83		0.84	0.82	0.90		0.83		0.90	0.83	0.99	0.90		0.90	0.99	**1.00**
Rec	0.96		0.99		0.96	0.99	0.95		0.94		0.95	0.94	1.00	0.91		0.91	1.00	**1.00**
F1	0.89		0.90		0.89	0.89	0.92		0.88		0.92	0.88	0.99	0.90		0.91	0.99	**1.00**
